# Genomic history of the Italian population recapitulates key evolutionary dynamics of both Continental and Southern Europeans

**DOI:** 10.1186/s12915-020-00778-4

**Published:** 2020-05-22

**Authors:** Marco Sazzini, Paolo Abondio, Stefania Sarno, Guido Alberto Gnecchi-Ruscone, Matteo Ragno, Cristina Giuliani, Sara De Fanti, Claudia Ojeda-Granados, Alessio Boattini, Julien Marquis, Armand Valsesia, Jerome Carayol, Frederic Raymond, Chiara Pirazzini, Elena Marasco, Alberto Ferrarini, Luciano Xumerle, Sebastiano Collino, Daniela Mari, Beatrice Arosio, Daniela Monti, Giuseppe Passarino, Patrizia D’Aquila, Davide Pettener, Donata Luiselli, Gastone Castellani, Massimo Delledonne, Patrick Descombes, Claudio Franceschi, Paolo Garagnani

**Affiliations:** 1 Laboratory of Molecular Anthropology & Centre for Genome Biology, Department of Biological, Geological and Environmental Sciences, University of Bologna, Bologna, Italy; 2 Interdepartmental Centre Alma Mater Research Institute on Global Challenges and Climate Change, University of Bologna, Bologna, Italy; 3 Department of Archaeogenetics, Max Planck Institute for the Science of Human History, Jena, Germany; 4 Department of Molecular Biology in Medicine, Civil Hospital of Guadalajara “Fray Antonio Alcalde” and Health Sciences Center, University of Guadalajara, Guadalajara, Jalisco Mexico; 5 Nestlé Research, EPFL Innovation Park, Lausanne, Switzerland; 6 Current Address: Lausanne Genomic Technologies Facility, University of Lausanne, Lausanne, Switzerland; 7 IRCCS Bologna Institute of Neurological Sciences, Bologna, Italy; 8 Department of Experimental, Diagnostic, and Specialty Medicine, University of Bologna, Bologna, Italy; 9 Applied Biomedical Research Center (CRBA), S. Orsola-Malpighi Polyclinic, Bologna, Italy; 10 Functional Genomics Laboratory, Department of Biotechnology, University of Verona, Verona, Italy; 11 Current Address: Menarini Silicon Biosystems SpA, Castel Maggiore, Bologna, Italy; 12 Geriatric Unit, Fondazione Ca’ Granda, IRCCS Ospedale Maggiore Policlinico, Milan, Italy; 13 Department of Experimental and Clinical Biomedical Sciences “Mario Serio”, University of Florence, Florence, Italy; 14 Department of Biology, Ecology and Earth Sciences, University of Calabria, Rende, Italy; 15 Department of Cultural Heritage, University of Bologna, Ravenna, Italy; 16 Department of Applied Mathematics, Institute of Information Technology, Lobachevsky University of Nizhny Novgorod, Nizhny Novgorod, Russia; 17 Clinical Chemistry, Department of Laboratory Medicine, Karolinska Institutet at Huddinge University Hospital, Stockholm, Sweden

**Keywords:** Italian population, Whole-genome sequences, Demographic inference, Polygenic adaptation, Evolutionary medicine

## Abstract

**Background:**

The cline of human genetic diversity observable across Europe is recapitulated at a micro-geographic scale by variation within the Italian population. Besides resulting from extensive gene flow, this might be ascribable also to local adaptations to diverse ecological contexts evolved by people who anciently spread along the Italian Peninsula. Dissecting the evolutionary history of the ancestors of present-day Italians may thus improve the understanding of demographic and biological processes that contributed to shape the gene pool of European populations. However, previous SNP array-based studies failed to investigate the full spectrum of Italian variation, generally neglecting low-frequency genetic variants and examining a limited set of small effect size alleles, which may represent important determinants of population structure and complex adaptive traits. To overcome these issues, we analyzed 38 high-coverage whole-genome sequences representative of population clusters at the opposite ends of the cline of Italian variation, along with a large panel of modern and ancient Euro-Mediterranean genomes.

**Results:**

We provided evidence for the early divergence of Italian groups dating back to the Late Glacial and for Neolithic and distinct Bronze Age migrations having further differentiated their gene pools. We inferred adaptive evolution at insulin-related loci in people from Italian regions with a temperate climate, while possible adaptations to pathogens and ultraviolet radiation were observed in Mediterranean Italians. Some of these adaptive events may also have secondarily modulated population disease or longevity predisposition.

**Conclusions:**

We disentangled the contribution of multiple migratory and adaptive events in shaping the heterogeneous Italian genomic background, which exemplify population dynamics and gene-environment interactions that played significant roles also in the formation of the Continental and Southern European genomic landscapes.

## Background

To date, several studies aimed to elucidate the genetic legacy of modern European populations, having accumulated evidence that it has been shaped by complex prehistoric and historical processes resulting from the contact between groups with appreciably different ancestries [1–8]. In particular, the genetic makeup of the current European meta-population was found to be characterized by a clinal distribution of variation, with subtle divergence observable especially between people from Continental and Southern Europe [9, 10]. This pattern is recapitulated uniquely by genetic variation distributed along the Italian Peninsula [11–13], suggesting that the dissection of demographic and evolutionary events occurred in this area may improve the understanding of key population dynamics and gene-environment interactions having contributed to the formation of the present-day European genomic landscape [14–17].

Several studies relying on the analysis of uniparental markers [11–14] or genome-wide autosomal polymorphisms [15–17] already drew a detailed picture of the fine-scale genetic structure of the Italian population, as well as of the linguistically and/or geographically isolated communities present on the Italian territory [18–21]. These research efforts provided also intriguing clues about the demographic history of the ancestors of present-day Italians. For instance, small pre-Neolithic contributions were proposed to have survived in the Italian Y chromosome and autosomal gene pools [13, 16, 17]. Nevertheless, the appreciable frequency of some maternal lineages especially in Southern Italy suggested a link with populations from the Caucasus and the Levant, which predates the Neolithic and may support the role of this area as a refugee during the Last Glacial Maximum (LGM) [14, 22]. Despite that, Neolithic and post-Neolithic population movements are supposed to have predominantly shaped modern patterns of Italian variation. In fact, the genetic distinctiveness of Sardinians with respect to peninsular Italians [23–25] was interpreted as a relic signature of an Early Neolithic European genomic background that might have been preserved especially in such a population due to its isolation to subsequent large-scale migrations [16, 26, 27]. Moreover, the establishment of a north-west to south-east cline of Y chromosome variation along the peninsula was proposed to date back to two antiparallel migration waves that brought the Neolithic in Southern Italy and the Adriatic coasts earlier than in the northern and Tyrrhenian regions [13, 28, 29]. A significant impact of Late Neolithic and Bronze Age demic processes on Italian Y chromosome and autosomal gene pools was further hypothesized [13, 17], along with subsequent influences especially on people from Northern Italy that may be related to events that occurred during the Roman Empire and the Middle Ages [15, 16]. Gene flow from the Near East instead seems to have affected mainly Central Italy and for a longer period than other regions of the peninsula [16]. Finally, Southern Italians were found to present genetic affinity with populations from the Eastern Mediterranean and particularly from Crete, Cyprus, and the Anatolian/Dodecanese islands [17], with people from Sicily also showing increased proportion of ancestry components likely introduced during the Arab occupation of the island [14, 16]. According to this picture, the ancestors of present-day Italians are supposed to have experienced an extraordinarily tangled history of migrations and gene flow, which is the main factor underlying the well-established cultural and genetic diversity of the Italian population, some of the most outstanding among those observable across the entire European continent [19].

Furthermore, due to the remarkable latitudinal range of the peninsula, which spans from the Alps to the core of the Mediterranean Sea, human groups who anciently spread along it were likely forced to cope with considerably different ecological, environmental, and climate conditions. As a result, the heterogeneous Italian genomic background may have represented a favorable substrate for the action of natural selection enabling the evolution of different local adaptations triggered by a variety of selective pressures [16]. Accordingly, despite being largely understudied, investigation of the adaptive history of the Italian people promises to pinpoint a valuable compendium of gene-environment interactions having played a relevant role in the evolution of European populations.

Nevertheless, previous studies focused on the genetic history of Italians mostly relied on inferences drawn from the analysis of single genetic systems (i.e., mitochondrial DNA and Y chromosome) or of moderate-to-high frequency autosomal single nucleotide polymorphisms (SNPs). This prevented to exhaustively investigate the full spectrum of variation observable in the Italian gene pool particularly underestimating the information associated with low-frequency and/or small effect size variants, which are insufficiently surveyed by SNP arrays. However, these typologies of alleles have been revealed as pivotal in determining patterns of fine-scale population structure [30–32] and as important genetic determinants of complex traits [33], including adaptive ones if considering a polygenic adaptation model that seems to be more realistic than those based on hard/soft selective sweeps [34–38].

To overcome these issues and to provide the as exhaustive as possible picture of the demographic and adaptive history of the ancestors of present-day Italians, we took advantage of high-coverage (90×) whole-genome sequence (WGS) data generated for 38 subjects native from different Italian regions. Building on the results from previous studies, we selected individuals potentially representative of the two genetically homogeneous population clusters (i.e., the northern and southern ones, respectively referred to as N_ITA and S_ITA) presenting the most distinct ancestry proportions and lying at the opposite ends of the cline of genetic variation observable along the peninsula [16]. This enabled us to infer dissimilar relationships of these main Italian groups with a large panel of modern and ancient Euro-Mediterranean populations providing novel evidence for the demographic processes having predominately left indelible traces in their genomes. Moreover, this approach disclosed new knowledge on the adaptive evolution of the ancestors of present-day Italians, by paving the way to the identification of previously undetected events of positive and balancing selection having mediated their biological adaptation to locally diverging ecological, environmental, and cultural contexts.

## Results

After the application of stringent quality control (QC) procedures (see the “Methods” section), we assembled a high-quality dataset including 38 Italian samples characterized for more than 17 million single nucleotide variants (SNVs). To confirm their self-reported ancestry from a genetic perspective, we performed a Procrustes analysis on a dataset including also genome-wide genotype data already available for 737 Italian individuals with known micro-geographical origins [16]. As expected, the sequenced samples clustered within the variability ranges of the previously identified N_ITA and S_ITA Italian population clusters [16], occupying diametrically opposed positions along the well-known north-south cline of Italian variation (Additional file 1: Figure S1) [16, 39–76].

### Setting Italians into the Euro-Mediterranean genomic landscape

To further test the membership of the sequenced individuals to distinguishable Italian population clusters and to frame them within the broad genomic landscape of populations from Continental and Southern Europe, Near East, and North Africa, we used literature WGS data [77] to create a “high-density Euro-Mediterranean dataset” including around seven million SNVs and we submitted it to the fineSTRUCTURE analysis.

**Fig. 1:**
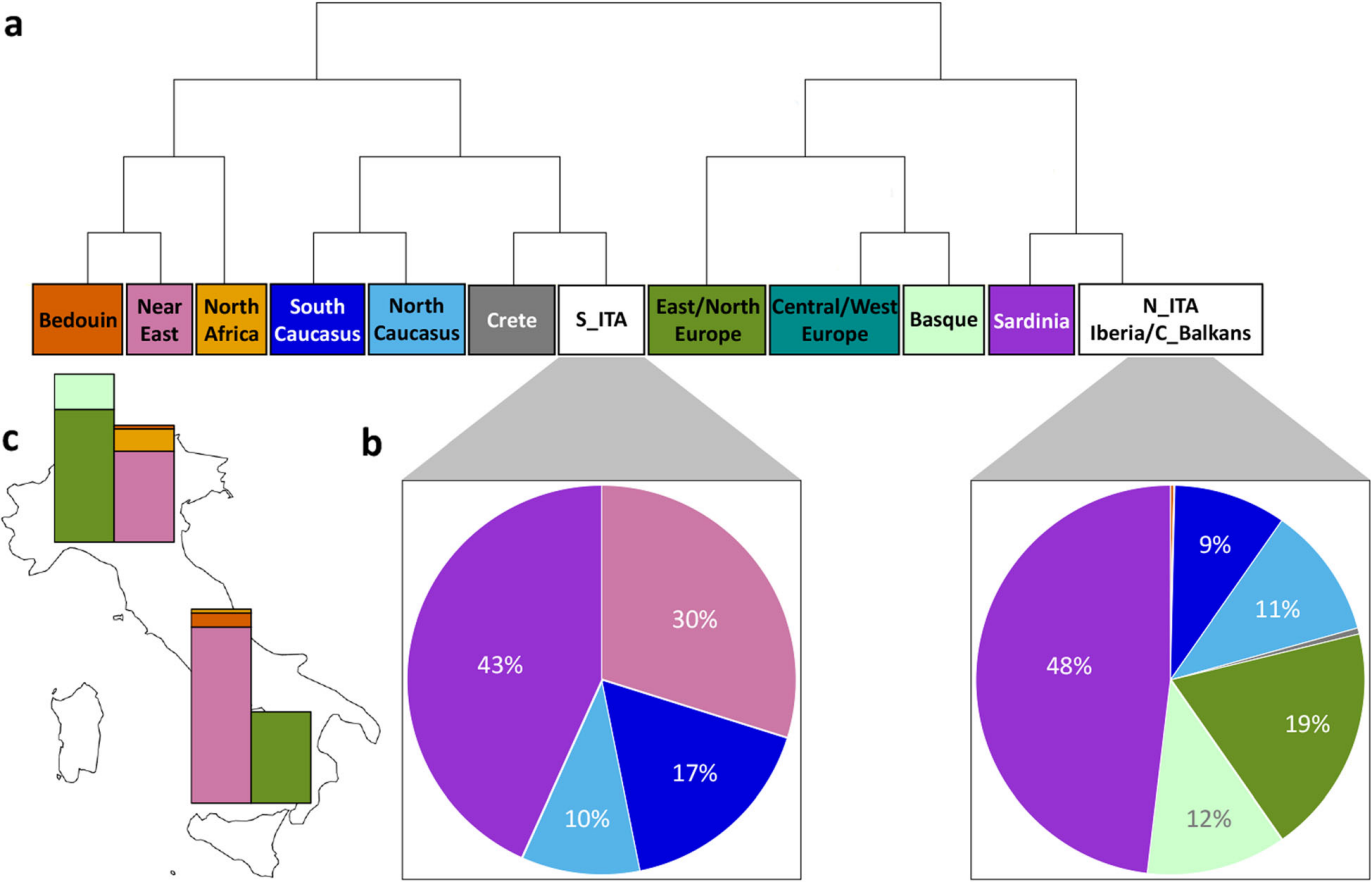
Clustering analysis and inference of admixture proportions performed on the “high-density Euro-Mediterranean dataset”. **a** fineSTRUCTURE hierarchical clustering reporting population clusters defined by collapsing branches of the obtained dendrogram that split with a posterior probability lower than 80%. N_ITA formed a cluster with Iberians and continental Balkan individuals from Bulgaria and Albania (C_Balkans). **b** Percentages of chromosome chunks shared between Italian and Euro-Mediterranean population clusters obtained with CHROMOPAINTER. Painting profiles showed in the pie charts are color-coded according to the palette used for fineSTRUCTURE clusters. **c** Ancestry proportions of the Italian population clusters inferred with the GLOBETROTTER pipeline from CHROMOPAINTER outputs. For each cluster, the bar on the left represents the major source of admixture, while the bar on the right represents the minor one. For details on the different subcomponents of these admixture sources, see Additional file 1: Table S1. To infer potentially different mixing proportions of N_ITA e S_ITA groups with respect to the other identified population clusters, all Euro-Mediterranean individuals were considered as recipients, while the two Italian groups were excluded from the donors. Admixture proportions showed in the bar charts are color-coded according to the palette used for the fineSTRUCTURE clusters

By considering only clusters splitting with a posterior probability above 80% (see the “Methods” section), Italian samples turned out to be located on two considerably divergent branches (*F*_st_ = 0.0021; *p* value < 10^−6^) of the dendrogram drawn from the obtained co-ancestry matrix (Fig. 1a). In detail, S_ITA subjects clustered apart from N_ITA ones and close to individuals from Crete, branching out from the node originating also the Northern Caucasian (i.e., North Ossetians, Chechens, Adygei, and Lezgins) and Southern Caucasian (i.e., Georgians, Abkhasians, Armenians, and Turks) clusters. Moreover, all of these groups further diverged from the node basal to Near Eastern (i.e., Bedouins, Palestinians, and Jordanians) and North African (i.e., Mozabites and Saharawi) populations. N_ITA samples instead formed a cluster that included also Iberian, Bulgarian, and Albanian individuals, branching out from the node leading also to Sardinians and considerably diverging from the Basques, as well as from the remaining European populations. These latter groups formed two distinct clusters: one made up of Central and Western Europeans (i.e., Hungarians, Czechs, Polish, French, Orcadians, and British people) and another one including populations from Eastern and Northern Europe (i.e., Russians, Estonians, Finnish, Norwegians, and Icelandic people) (Fig. 1a).

### Depicting patterns of recent admixture between Italian and Euro-Mediterranean populations

Sharing of chromosome chunks among individuals belonging to the identified population clusters was investigated with CHROMOPAINTER. Accordingly, both Italian groups were found to share similar proportions of DNA segments with Sardinians (N_ITA, 48%; S_ITA, 43%) and Northern Caucasian populations (~ 10%), which have been previously supposed to be suggestive respectively of Early Neolithic and Bronze Age contributions to the ancestral pan-European genetic background [7, 8], while presenting considerably different painting profiles for the rest of their genomes (Fig. 1b). In particular, S_ITA showed substantial sharing (30%) with Near Eastern populations, while this signature is completely absent in N_ITA. Moreover, S_ITA presented 17% of chromosome chunks in common with Southern Caucasian groups in contrast to the 9% observed for N_ITA, although this pattern might be influenced by the fact that populations from Southern Caucasus are genetically close to those from the Near East [77]. N_ITA finally turned out to share DNA segments also with Eastern and Northern European groups (19%) and the Basques (12%), differently from what was observed for S_ITA (Fig. 1b).

CHROMOPAINTER painting profiles were then used to infer admixture proportions of N_ITA and S_ITA with respect to the other Euro-Mediterranean clusters by calculating co-ancestry curves with the GLOBETROTTER method (see the “Methods” section). Admixture events involving a Northern European (and Basque in the case of N_ITA) gene pool and a Near Eastern/North African source of gene flow were found to have affected both Italian population clusters (Fig. 1c, Additional file 1: Figure S2). Nevertheless, N_ITA and S_ITA showed inverse proportions of these admixture sources, with respectively 59% and 32% of Northern European (and Basque in the case of N_ITA) contribution, coupled with 41% and 68% of Near Eastern and North African one (Fig. 1c, Additional file 1: Table S1). By considering 95% confidence intervals of the estimated admixture dates, these events of gene flow appeared to be temporally overlapping and overall ranged from 1.2 to 2 thousand years ago (kya), with those involving S_ITA being slightly shifted towards more recent times (Additional file 1: Table S2). In particular, in agreement with the results from previous studies [15, 16], we inferred gene flow from continental Europe to N_ITA as occurred especially at the end of the Roman Empire and during the Middle Ages, while Middle Eastern and North African contributions to the Italian gene pool were found to be concomitant with the Byzantine and Arab expansions in Central and Southern Italy. Nevertheless, rather than contributing novel ancestry factions, these admixture events may have played a role in reinforcing the differential distribution of ancient genetic components already present in the Italian groups, thus additionally shaping their differences in ancestry profiles.

### Exploring the ancient genetic legacy of Italian population clusters

To expand the inference of genetic ancestry shared between Italian population clusters and other Euro-Mediterranean groups far beyond the relatively recent timescale investigated by GLOBETROTTER analysis, we took advantage of the genome-wide data for 559 ancient DNA (aDNA) samples by assembling a “modern + aDNA dataset” (see the “Methods” section).

**Fig. 2:**
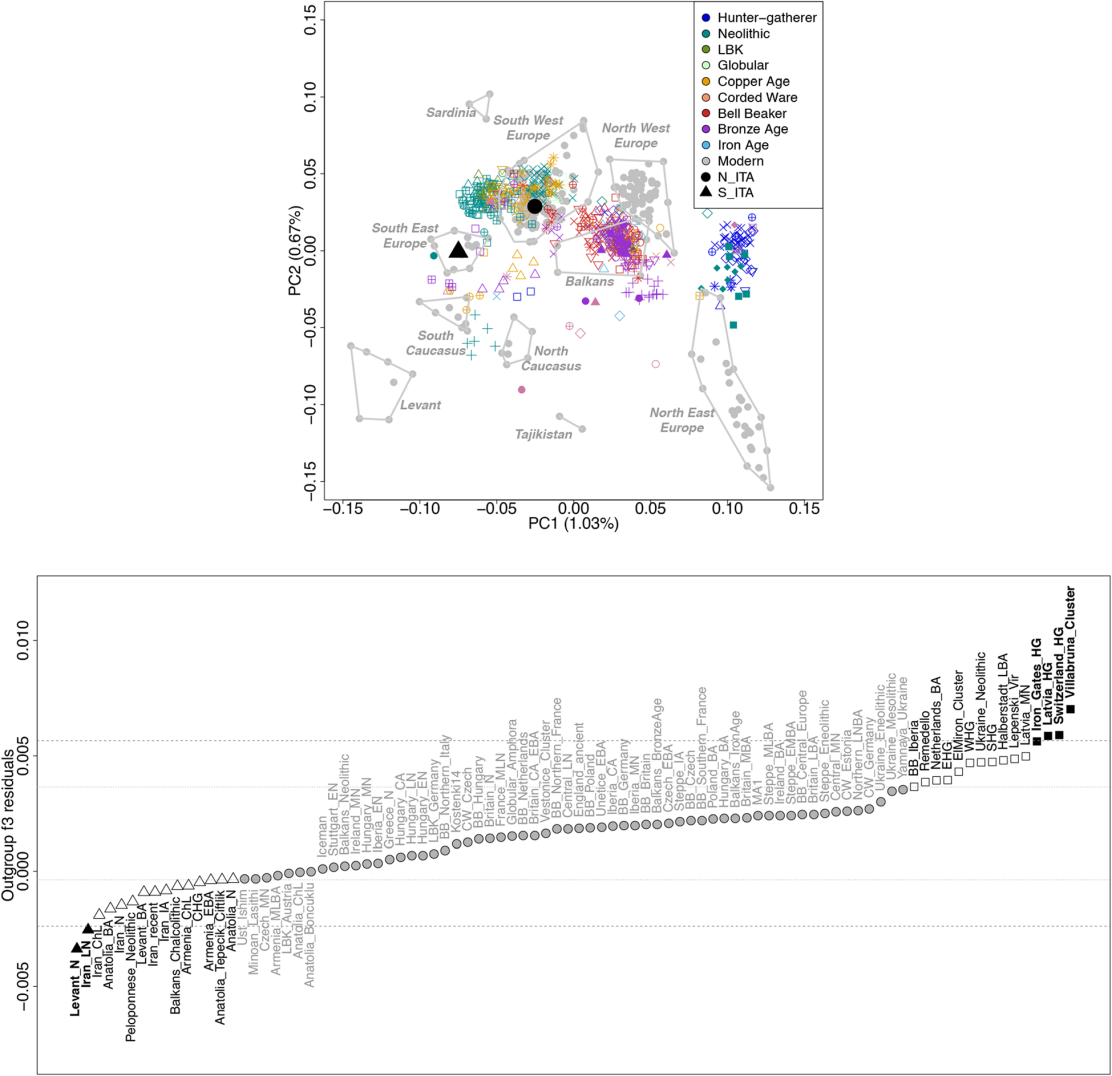
PCA and outgroup *f3* analysis performed on the “modern + aDNA dataset”. **a** PCA projecting variation of 559 ancient samples onto the genetic space defined by 239 individuals belonging to 40 modern Euro-Mediterranean populations. Modern samples are reported as gray dots, with the exception of centroids calculated for the N_ITA and S_ITA clusters (black dot and black triangle, respectively), while the main ancient groups are color-coded according to their temporal/cultural framework. For details on the populations of origin of ancient samples, see the legends in Additional file 1: Figure S3. **b** Distribution of outgroup *f*3 residuals between N_ITA and S_ITA clusters. Residuals were calculated as the difference in outgroup *f3* scores computed in the form *f3* (Han Chinese (CHB); X Italian population cluster, X ancient population cluster) to test in turn the N_ITA and S_ITA clusters against each ancient population group (reported on the x-axis). Residuals are reported from the most negative (i.e., suggesting closer affinity of ancient populations to S_ITA) to the most positive one (i.e., suggesting closer affinity of ancient populations to N_ITA); those exceeding one or two standard deviations (SDs) (indicated by dashed lines) from the mean of the obtained distribution are color-coded in white and black squares for N_ITA and in white and black triangles for S_ITA, respectively

Principal component analysis (PCA) projecting ancient variation onto the genetic space defined by modern populations suggested appreciably different ancestral contributions to the N_ITA and S_ITA groups (Fig. 2a, Additional file 1: Figure S3). In particular, N_ITA individuals, which clustered close to people from the Iberian Peninsula (IBS) within the bulk of modern southwestern Europeans, showed a particular affinity with Central European, Hungarian, and British Neolithic samples; Copper Age subjects from Hungary and the Balkans; a Corded Ware Czech remain; and Iberian and Hungarian individuals belonging to the Bell Baker culture. Moreover, the centroid of the N_ITA cluster lay in proximity to the Copper Age Northern Italian Remedello sample. Conversely, S_ITA subjects showed tight relatedness with modern southeastern European populations (e.g., Cretans and Greeks), along with Neolithic, Copper Age, and Bronze Age Anatolian samples; Minoan remains from Crete, Neolithic, and Bronze Age Levantine individuals; and Chalcolithic Iranians (Fig. 2a, Additional file 1: Figure S3).

Ancient samples were then grouped according to the patterns of genetic clustering pointed out by PCA, as well as by considering their archeological/temporal frameworks, and used to formally measure the shared genetic drift between them and present-day Italian population clusters by computing outgroup *f3* statistics. Moreover, the obtained outgroup *f3* scores were contrasted between N_ITA and S_ITA to search for potentially relevant differences in their ancestral genetic contributions (see the “Methods” section). Accordingly, the bulk of the calculated scores was found to be distributed along the diagonal line of an outgroup *f*3 biplot indicating overall overlapping of N_ITA and S_ITA genetic relationships with aDNA samples (Additional file 1: Figure S4). However, some remarkable differences (i.e., residuals) between N_ITA and S_ITA outgroup *f*3 statistics were observed as concerns specific ancient population groups (Fig. 2b). In particular, negative residuals suggesting closer affinity of aDNA samples to the S_ITA cluster were found to exceed one standard deviation (SD) from the mean of the obtained distribution when hunter-gatherers from the Caucasus, Neolithic, and Chalcolithic/Bronze-Age samples from Anatolia, Near East, Greece, and the Balkans were considered. Negative values even more outstanding (i.e., exceeding two SDs) were then observed in relation to the Levant and Iranian Neolithic samples. Conversely, positive residuals suggesting closer affinity of ancient populations to the N_ITA cluster and exceeding one SD were found by taking into account especially Iberian individuals belonging to the Bell Baker culture, the Copper Age Northern Italian Remedello specimen, and hunter-gatherer and Bronze Age samples from Central and Eastern Europe. Moreover, the most outstanding positive values (i.e., exceeding two SDs) were obtained when modern Italians were tested against hunter-gatherer groups from the Continental Europe and the Villabruna clusters (Fig. 2a).

### Inferring *N*_e_ histories and divergence time between Italian population clusters

**Fig. 3:**
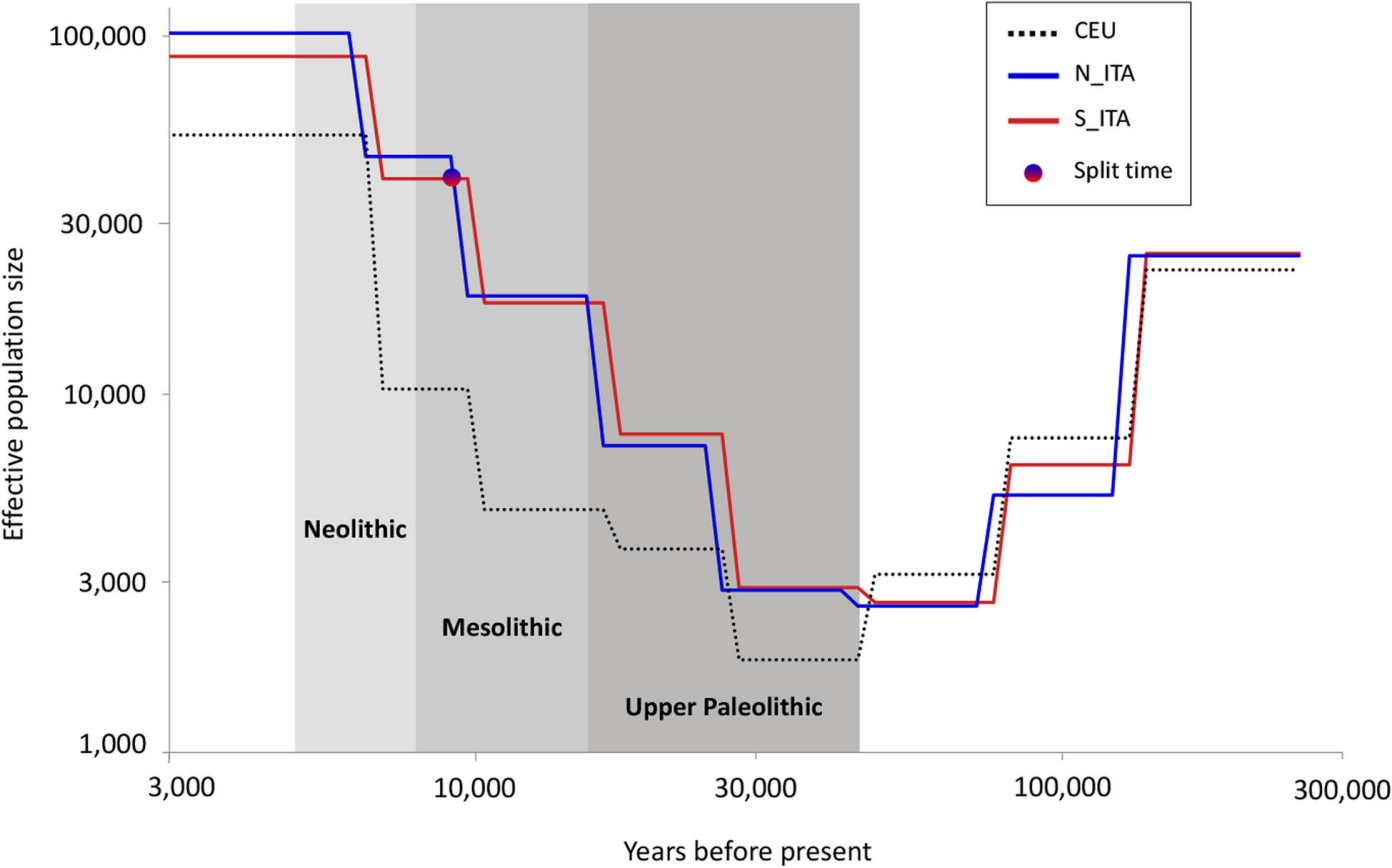
Coalescent-based inference of population size histories and genetic split time by means of the SMC++ method. Population size trajectories of N_ITA and S_ITA groups were estimated by setting 150 generations as the most recent time point for population size inference and 10 spline knots to anchor the size history and were compared to that inferred for CEU. The shaded boxes indicate Upper Paleolithic, Mesolithic, and Neolithic periods

To narrow down the time frame of the main population dynamics having contributed to the observed differentiation between N_ITA and S_ITA clusters, their population size histories and genetic divergence were explicitly modeled by means of the sequential Markov coalescent + plenty of unlabeled samples (SMC++) method. Changes in N_ITA and S_ITA effective population sizes (*N*_e_) were thus inferred and compared to those observed for a population of Northern and Western European ancestry (CEU). Accordingly, ancestors of all groups were found to have experienced a steep decline in *N*_e_ since approximately 130 kya and until 70–50 kya, which plausibly reflects the strong bottleneck suffered by ancestral non-African populations during the Out-of-Africa migrations of modern humans. The demographic expansion that characterized all European groups since around 30 kya was then observed, with the ancestors of Italians having maintained consistently higher *N*_e_ with respect to those of CEU (Fig. 3) in agreement with what was previously observed when comparing the Southern and Continental European populations [78]. Moreover, when the genetic distinction between Italian clusters was modeled as a function of time according to an idealized two-population split scenario with no post-divergence gene flow, appreciable differentiation between N_ITA and S_ITA was found to emerge since around 9 kya (Fig. 3).

### Disentangling the action of positive and balancing selection on the Italian genomes

Genomic signatures ascribable to the action of positive and balancing selection on N_ITA and S_ITA ancestors were detected by computing the derived intra-allelic nucleotide diversity (DIND) and the number of segregating sites by length (nSL) scores, as well as by applying the BALancing selection LikElihood Test (BALLET). Genome-wide distributions obtained for these statistics were then used as input for gene network analyses aimed at testing the adaptive evolution of the Italian population groups under a model as close as possible to that of polygenic adaptation (see the “Methods” section). To focus on local adaptations that specifically characterize the ancestors of present-day Italians, we replicated these analyses on WGS data for the IBS and CEU populations, and we filtered out signatures of natural selection shared between them and the Italian clusters. In fact, although we cannot rule out the possibility that gene flow from other European and Mediterranean human groups has contributed to the evolution of these biological adaptations, this filtering approach enabled us to shortlist those selective signatures that are more plausibly ascribable to a combination of nature, duration, and intensity of the selective pressures that was peculiar of the Italian Peninsula.

According to the DIND test, *RIMS2* and *PCLO* genes involved in insulin exocytosis (Additional file 1: Supplementary Results) were found to be subjected to positive selection in both N_ITA and S_ITA clusters (Additional file 1: Figure S5, Table S3). A gene network belonging to the *Mucin type O-glycan biosynthesis* pathway and formed by loci encoding for mucins, a family of glycosylated proteins that constitute the main protective barrier on mucosal surfaces and cellular membranes by preventing pathogens binding by steric hindrance [61], was instead supposed to have adaptively evolved only in S_ITA (Additional file 1: Figure S6, Table S3, Supplementary Results).

According to the nSL test, a gene network ascribable to the *insulin secretion* pathway, but made up of different loci with respect to those pointed out by DIND scores, characterized the N_ITA cluster (Additional file 1: Figure S5, Table S4). Among these genes, *ADCY2*, *ADCY3*, *ADCY9*, and *GNAS* are known to play a role in the regulation of lipolysis at the level of the adipose tissue, thermogenesis, and glucagon signaling (Additional file 1: Supplementary Results), with especially adenylate cyclase (ADCY) genes showing the largest number of connections in the network and participating to the *longevity regulating* pathway as well. Moreover, variants at two loci belonging to such a network and encoding for components of calcium voltage-gated channels (i.e., *CACNA1C* and *CACNA1D*) were previously reported to be involved in the development of type II diabetes (T2D) (Additional file 1: Supplementary Results). nSL results obtained for S_ITA corroborated those based on the DIND statistics as regards a gene network belonging to the *mucin type O-glycan biosynthesis* pathway (Additional file 1: Figure S6, Table S4) and further indicated genes from the *basal cell carcinoma* pathway as subjected to positive selection (Additional file 1: Figure S7, Table S4). Interestingly, most of these latter loci encode for frizzled G protein-coupled receptors (FZD) and Wnt glycoproteins that play a role in melanogenesis and participate in the *mTOR signaling* pathway as well (Additional file 1: Supplementary Results).

Events of balancing selection at two different gene networks were inferred for both the Italian population clusters (Additional file 1: Table S5). In detail, a network was found to be composed by several ADCY genes, some of which (i.e., *ADCY2* and *ADCY9*) were the same putatively subjected also to positive selection in N_ITA, as well as by mitogen-activated protein kinase (MAPK) genes (i.e., *MAPK1/ERK*, *MAPK8/JNK*, and *MAPK11/P38*). These loci are known to play a role especially in the *longevity regulating* and *FoxO signaling* pathways (Additional file 1: Supplementary Results). The second network was instead made up of glycerol phosphate acyltransferase (*GPAT/AGPAT/MBOAT*), diacylglycerol kinase (*DGKA*), and lipid phosphatase (*LPIN/PLPP*) genes regulating the metabolism of glycerolipids, along with phospholipase (e.g., *PLA2G*/*PLB*) loci involved especially in the arachidonic acid metabolism (Additional file 1: Supplementary Results). Two gene networks were found to characterize exclusively the N_ITA group, being composed of aldehyde dehydrogenase (ALDH) genes important for glycolysis/gluconeogenesis and of protein kinase C (*PRKC*) and phospholipase (*PLCG/PLCB*) loci participating to the *AGE-RAGE signaling in diabetic complications*, *glucagon signaling*, and *insulin resistance* pathways (Additional file 1: Table S5). Finally, a further network turned out to be subjected to balancing selection in the S_ITA cluster, including PLA2G genes involved in the metabolism of arachidonic acid and *MAPK1* and *GRM1* loci that play a role in the *FoxO signaling* pathway (Additional file 1: Table S5, Supplementary Results).

## Discussion

The clinal distribution of human genetic variation across Europe and the subtle divergence between groups from northern and southern regions of the continent are uniquely recapitulated at a micro-geographic scale by patterns of population structure observable along the Italian Peninsula [11–13, 16, 21]. To date, remarkable efforts have elucidated important aspects of the demography of the ancestors of modern Italians, which have contributed to their heterogeneous genetic background [14–19, 21, 22]. However, constraints imposed by the use of uniparental markers or of common autosomal SNPs affected the inferences drawn by these researches. Moreover, just a few studies attempted to complete the picture of the Italian genetic history with the investigation of local adaptations evolved by ancestral populations distributed along the peninsula in response to a wide range of environmental conditions [16, 23]. Additionally, none of them relied on data useful to test a model of polygenic adaptation mediated by natural selection slightly affecting many genes involved in the same biological function, but individually contributing a limited phenotypic effect, which has recently emerged as one of the predominant mechanisms of adaptive evolution of the human genome [35, 38].

In the attempt to overcome these issues, we aimed at depicting the demographic and adaptive history of the ancestors of present-day Italians by taking advantage of high-coverage WGS data. For this purpose, we first compared the examined genomes with genome-wide genotypes already available for the overall Italian population via a Procrustes analysis, demonstrating that they are representative of the two genetically homogeneous clusters (i.e., N_ITA and S_ITA) corresponding to the edges of the cline of Italian variation (Additional file 1: Figure S1). Moreover, fineSTRUCTURE clustering pointed to an appreciable divergence of these Italian groups (Fig. 1a), which is further supported by a low but highly significant genome-wide estimate of genetic differentiation (*F*_st_ = 0.0021; *p* value < 10^−6^). Previous studies have proven that, with the exception of Sardinians, N_ITA and S_ITA clusters encompass the most distinct ancestry components detectable at a considerable frequency in the Italian population and that, conversely, people from Central Italy present variable degree of admixture between them, but no additional private ancestry fractions [13, 15–17]. Therefore, the assembled WGS dataset enabled us to draw demographic and adaptive inferences according to a reliable approximation of the full spectrum of genetic components observable in the entire Italian gene pool.

### Late Glacial, Neolithic, and Bronze Age demographic processes left indelible signatures in the Italian genomes

Our fineSTRUCTURE analysis further suggested that divergence between Italian clusters was reflected by a wide genetic Mediterranean “continuum” involving S_ITA and populations from Crete and the Caucasus as opposed to the affinity of N_ITA with groups from Continental Balkans (e.g., Bulgaria and Albania) (Fig. 1a). As for S_ITA, this peculiar pattern was recently proposed to be ascribable to Neolithic and Bronze Age contributions to the local gene pool originating from the Near East and the Caucasus. In particular, the Caucasus was identified as the potential source of a Bronze Age population movement that impacted Southern Italy approximately at the same time but independently from the well-known steppe-related migrations that occurred in Continental Europe. Clear marks of the latter demographic process were instead observed in Northern Italy, as well as in Central and Northern Balkans [17]. The results from the analysis of residuals calculated by contrasting N_ITA and S_ITA outgroup *f3* statistics and using a large panel of aDNA samples are consistent with the hypothesis mentioned above. In fact, increased shared genetic ancestry with Chalcolithic/Bronze Age and, especially, Neolithic remains from Anatolia, Armenia, Near East, and Greece was inferred for S_ITA with respect to N_ITA, with the largest residuals pointing to the relationships of S_ITA with populations from Iran and the Levant dating back to the Neolithic (Fig. 2b). These findings confirm the early positioning of Southern Italy at one of the westernmost edges of the extensive Mediterranean corridor that mediated the diffusion of farming from Southeastern Europe [5, 28, 79] and suggested Neolithic processes having left some of the most substantial traces (e.g., in terms of Anatolian-Neolithic-related and Caucasus hunter-gatherer ancestries) in the genetic background of S_ITA people. Moreover, they suggested that subsequent Chalcolithic/Bronze Age population movements having influenced the S_ITA gene pool have plausibly originated from Southern Caucasus and Anatolia and reached the Italian Peninsula through a Mediterranean route [7]. In addition to gene flow that occurred during historic times along the same path (Fig. 1c, Additional file 1: Tables S1-S2), this ancient connection contributes to explain also the patterns of haplotype sharing with present-day populations from the Near East and Southern Caucasus that were observed predominantly for S_ITA (Fig. 1b).

On the contrary, a more substantial ancestry shared by N_ITA with Western European remains dated to the Copper Age or associated with the Bell Baker complex was observed along with their increased affinity to the Central and Eastern European Bronze Age samples. Again, this is concordant with N_ITA chromosome painting profiles and ancestry proportions shared with modern groups such as the Basques and Eastern/Northern Europeans (Fig. 1b, c; Additional file 1: Table S1). Interestingly, signatures ascribable to relationships with considerably more ancient groups, including Eastern, Western, and Scandinavian hunter-gatherers and samples belonging to the Late Glacial “El Miron Cluster” also emerged, with the largest outgroup *f3* residuals being associated with hunter-gatherer specimens from the Balkans, Latvia, and Switzerland, as well as with the post-Ice Age “Villabruna cluster” (Fig. 2a). Contrarily to what is supposed for Sardinians [27], we speculate that this pattern is only partially ascribable to a direct link of present-day N_ITA with local Upper Paleolithic groups. Instead, this is in line with the hypothesis that population movements that involved the Italian Peninsula during and after the Neolithic have replaced a great part of local Paleolithic genetic backgrounds [13, 16, 17]. Accordingly, the observed affinity with hunter-gatherer samples might be likely due to the resurgence of genetic components proper of the early European founder population because of demographic processes that occurred during the Late Glacial and, particularly, the Bronze Age. This is suggested by N_ITA affinity with “El Miron Cluster,” which is dated to around 19–14 kya and was found to attest a post-Ice Age re-expansion from southwestern European refugia of an ancestry fraction that was widespread all over Europe between 34 and 26 kya [4]. The close relationship with the “Villabruna Cluster” might instead reflect the impact that the diffusion of the Epigravettian culture exerted on the ancestral N_ITA gene pool since the end of the LGM [4]. Finally, groups migrated from the Eurasian Steppe during the Early Bronze Age, such as Yamnaya pastoralists, have been previously demonstrated to present substantial pre-Neolithic ancestry fractions in addition to their peculiar steppe-related genetic component [2, 3, 80] Consequently, these population movements are supposed to have contributed to raise again the Eastern hunter-gatherer ancestry in Western Europeans since around 4.5 kya, as testified by several remains belonging to the Bell Baker complex and including the Iberian ones that showed increased shared genetic drift with N_ITA [8].

Overall, the distinct ancestry composition described for N_ITA and S_ITA clusters fits well also with the demographic scenario depicted by modeling their ancient and recent population history with the coalescent-based SMC++ method (Fig. 3). The seemingly higher *N*_e_ inferred for S_ITA with respect to N_ITA until the beginning of the Late Glacial might be compatible with the hypothesis of a refugee role played by Southern Italy during the LGM (see also the paragraph below about climate-mediated adaptations) [14, 22, 28]. However, it is not possible to evaluate the actual statistical significance of this subtle *N*_e_ difference, at least as concerns the period that predates the inferred population split time. Moreover, this pattern might be also ascribable to the more substantial level of gene flow from diverse populations experienced by S_ITA with respect to N_ITA, as proposed to explain the differences in *N*_e_ observed between Southern and Continental European groups [78]. More interestingly, appreciable genetic differentiation between N_ITA and S_ITA can be approximately dated back to just after the end of the LGM (Fig. 3), if we consider that the obtained population split time (i.e., 9 kya) represents a rough underestimate due to a clear violation of the assumption of negligible post-divergence gene flow between clusters made by the SMC++ model. This is thus in line with a scenario assuming that the Late Glacial demographic processes described above have represented the first step in the cascade of events that differentially shaped the gene pool of present-day N_ITA and S_ITA groups.

### Climate-mediated adaptive evolution at insulin-related genes especially in Northern Italy

Both selection scans performed to test for the occurrence of positive and balancing selection suggested a complex pattern of adaptive evolution at insulin-related genes in the Italian people.

In detail, selective events able to modulate insulin exocytosis from pancreatic beta cells were supposed to have occurred in the common ancestors of N_ITA and S_ITA clusters (Additional file 1: Figure S5, Table S3, Supplementary Results). Events of positive selection presumably more recent were instead found to characterize exclusively people from N_ITA, being distributed among ten genes that play a role at different levels of the signaling cascade leading to insulin secretion and that regulate key processes contributing to glucose homeostasis (Additional file 1: Figure S5, Table S4, Supplementary Results). Interestingly, the most pervasive signature of selection was observed at ADCY genes (especially *ADCY3*), which are fundamental for controlling thermogenesis [45] and adiposity [46, 47] and have been proven to modulate susceptibility to T2D and obesity (Additional file 1: Supplementary Results). In line with these findings, analyses testing for balancing selection pointed to adaptive events specific of the N_ITA cluster and mediated by ALDH genes involved in glycolysis and gluconeogenesis or by PRKC and PLCG/PLCB loci playing a role in pathological mechanisms underlying insulin resistance and the onset of diabetic complications (Additional file 1: Table S5).

**Fig. 4:**
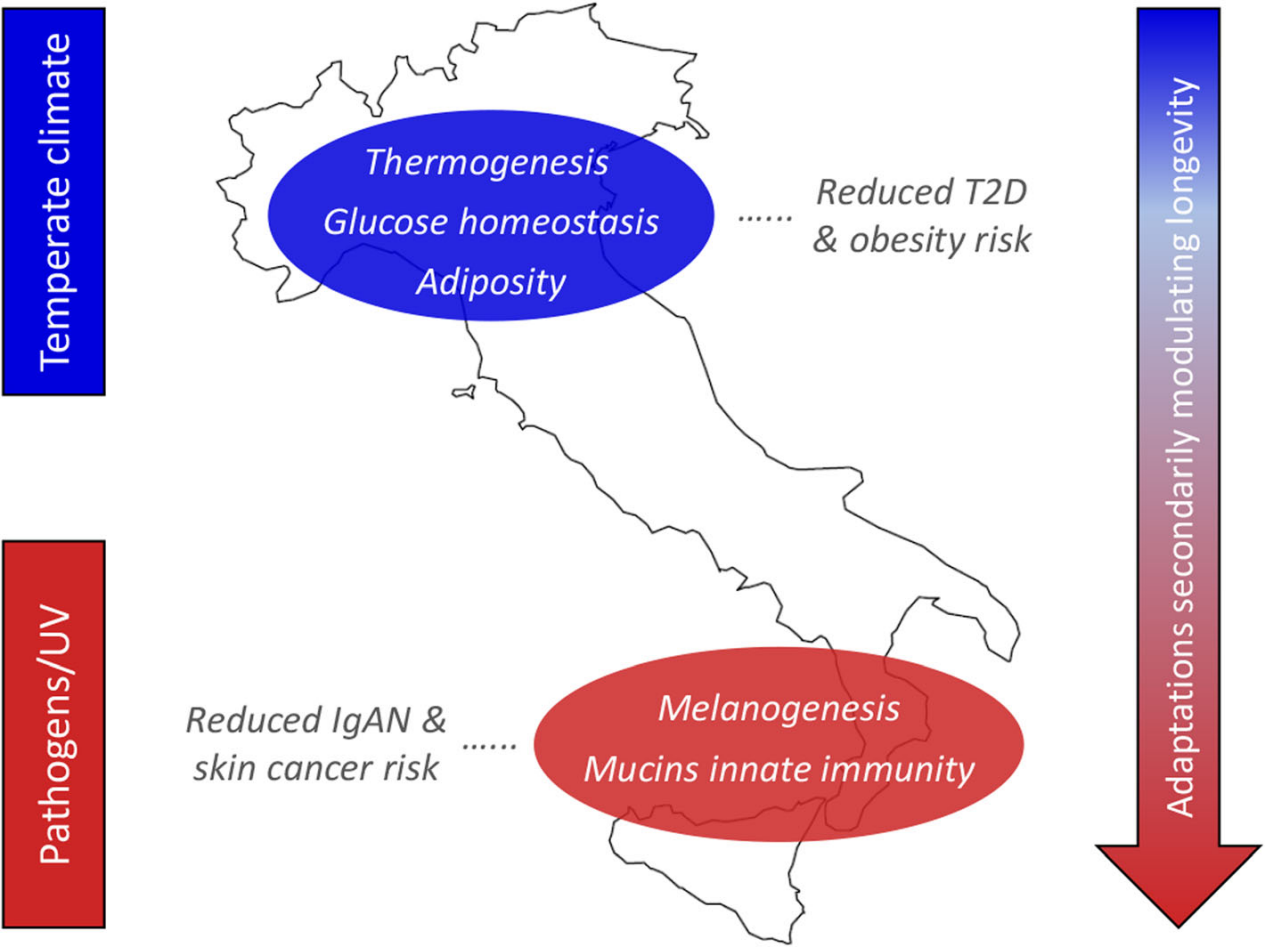
Adaptive events evolved by ancestors of N_ITA/S_ITA clusters and their health implications for present-day Italians. The putative selective pressures having plausibly prompted local adaptations are displayed on the left, while biological processes subjected to natural selection are reported on the map along with their impact on present-day disease susceptibility. Distribution of biological adaptations having the potential to modulate the longevity phenotype (e.g., involving the *mTOR signaling*, *arachidonic acid metabolism*, and *FoxO signaling* pathways) in the overall Italian population, but especially in people from Southern Italy, is represented by the arrow on the right. Putative selective pressures, biological processes, and distribution of adaptations potentially modulating longevity are color-coded as follows: N_ITA, blue; S_ITA, red

According to this body of evidence, we can speculate that climate- and tightly linked dietary-related selective pressures have presumably played a role in determining the described selection signatures (Fig. 4). The few ones shared between N_ITA and S_ITA clusters might indeed represent a legacy ascribable to the retreating of human groups distributed along the peninsula towards Central/Southern Italian refuge areas during the LGM [14, 22, 28]. There, northern and southern ancestral populations likely admixed and lived in forest-steppe habitats for around 10,000 years. This period was long enough to have possibly triggered optimization of energy metabolism in response to a cold environment in which animal-based high-energy/high-fat diets represented the main nutritional resource, as testified by isotope analyses on archeological records ascribable to the Gravettian and Epigravettian cultures [81]. This hypothesis is in agreement with evidence pointing to most of the adaptive events inferred so far for populations of Western European ancestry being dated to the LGM and correlating with environmental variables that suggest climate cooling and short-term temperature instability as some of the main selective pressures [82, 83].

With progressive climate warming during the Late Glacial, some groups moved back from refuge areas to repopulate the Northern Italian regions and, differently from populations expanding southwards who soon experienced again a Mediterranean climate, they continued to be subjected to selective pressures similar, although less extreme, to those acting during the LGM. For several other millennia, these people had to cope with a temperate climate characterized by cold winter seasons and have been more affected than Southern Italian groups by the climate changes that occurred in Continental Europe since the Bronze Age until recent historical times [84–86]. Although we cannot rule out the possibility that recent and differential gene flow from populations exposed to diverse environmental conditions contributed to exacerbate the differentiation of selection signatures observed between N_ITA and S_ITA groups, the climate picture described above has the potential to have represented a non-negligible factor in the evolution of more pervasive selective events by the ancestors of N_ITA, which extend beyond the simple regulation of insulin secretion to biological pathways able to modulate cell sensitivity to it, along with the metabolism of the adipose tissue and the expression of genes promoting thermogenesis (Fig. 4). This adaptive scenario fits well also with the picture of early differentiation between N_ITA and S_ITA clusters revealed by the SMC++ analysis, which become appreciable just since a few thousand years after the end of the LGM (Fig. 3). Interestingly, having targeted genes whose dysfunction is known to play a role in the development of T2D and/or obesity, most of the inferred N_ITA-specific signatures of positive and balancing selection seem to be ascribable to evolutionary events with potential biomedical relevance. For instance, adaptive evolution at these loci might have contributed to make people from Northern Italy less prone to develop such diseases even in the challenging nutritional environment imposed by modern lifestyles (Fig. 4). This is in line with the values of T2D incidence almost reduced by half in N_ITA with respect to S_ITA [87] and may further support recent attention drawn by our best candidate gene (i.e., *ADCY3*) as a promising target for the development of anti-obesity drugs [88].

### Pathogens and solar radiation may have triggered adaptations peculiar to Southern Italy

When considering adaptive events specific to S_ITA, genes encoding for mucins that prevent pathogens binding at the level of mucosal surfaces and loci participating in melanogenesis emerged as putative targets of positive selection (Fig. 4, Additional file 1: Figure S6-S7, Tables S3-S4).

Among mucin genes, *C1GALT1* represented the central node of the two identified gene networks (Additional file 1: Supplementary Results), and several genome-wide association studies previously reported a correlation of some of its variants to immunoglobulin-A nephropathy (IgAN), which is the most common human kidney inflammation [57]. Interestingly, epidemiologic data highlighted a considerably higher IgAN prevalence in Northern Italian regions than in Southern Italy [58]. According to this picture, and because several microorganisms are known to have evolved chemical strategies aimed at enzymatically inactivating mucins to elude mucosal/cellular barriers [57], we can hypothesize that some adaptive events that possibly occurred in response to these pathogens may have contributed to reduced S_ITA susceptibility to IgAN (Fig. 4). Among microorganisms able to inactivate mucins, *Pseudomonas aeruginosa*, the parasitic amoebozoan *Entamoeba histolytica*, and the proteobacterium *Burkholderia cepacia* present a geographical distribution that correlates negatively to that of IgAN and positively to environmental temperature (Additional file 1: Supplementary Results). Therefore, we can speculate that infections by these pathogens or by closely related species might have been more frequent in the past in Southern Italian regions than in northern ones, having potentially represented selective pressures able to trigger adaptive evolution of mucin genes in the ancestors of S_ITA.

Environmental conditions characterized by a mean value of annual solar radiation nearly double with respect to Northern Italy [89] might have played a role in the evolution of S_ITA-specific selection signatures at FZD/Wnt genes that are involved in melanogenesis (Fig. 4). In fact, being responsible for basal and ultraviolet (UV)-induced melanin production, melanocytes expressing these genes represent a frontline defense against harmful UV-B radiation. FZD genes found to have adaptively evolved in S_ITA act as receptors of Wnt protein ligands that showed comparable selection signatures and regulate the expression of the microphthalmia-associated transcription factor (*MITF*) [90]. By controlling pigmentation genes (e.g., *TYR*, *TYRP1*, and *TYRP2*), *MITF* is the main modulator of melanogenesis in response to environmental stimuli and was also proposed to exert an oncogenic role in several skin cancers [91]. This might explain the involvement of the identified FZD/Wnt genes under selection in the *basal cell carcinoma* pathway. Overall, these selective events could have mediated adaptations of S_ITA ancestors aimed at preventing skin micronutrient photodegradation and/or impairment of sweat gland-mediated thermoregulation due to UV damage [92]. Because substantial UV exposure represents the main risk factor for developing basal cell carcinoma and other types of skin malignancies, these adaptive mechanisms might have also indirectly contributed to reduce the predisposition of modern S_ITA to such diseases (Fig. 4). This hypothesis seems to be in agreement with the almost halved incidence of melanomas reported for Southern Italian regions with respect to northern ones [93].

### Pleiotropic adaptive events potentially modulating longevity in the Italian population

Several selection signatures observed for the overall Italian population, but resulting more pronounced in S_ITA, pointed to adaptive events mediated by biological processes that are known to play a role also in the achievement of the longevity phenotype (Fig. 4, Additional file 1: Table S5). Interestingly, this is in line with recent findings showing that Italian centenarians genetically cluster with people from Central/Southern Italy regardless of their micro-geographic origins [76]. Among the most relevant signatures, we emphasize S_ITA-specific positive selection at FZD/Wnt genes that take part in the *mTOR signaling* pathway as well. Overall, variants at loci belonging to this pathway have been demonstrated to be able to delay age-related diseases and/or to directly influence longevity even in the human species [67] (Additional file 1: Supplementary Results).

Identification of footprints of balancing selection at genes involved in the metabolism of arachidonic acid complements previous findings obtained for the general Italian population and for centenarians from the peninsula [16, 76] (Additional file 1: Supplementary Results). The emerging picture suggests that these adaptive events may have evolved in response to specific pathogens and secondarily maintained in the Italian gene pool alleles useful to contrast the side effects of modern pro-inflammatory diets, thus contributing to longevity [76]. Balancing selection was found to have targeted also several genes involved in the FoxO signaling, which provides monitoring of stress stimuli, such as dietary restriction, absence of insulin or insulin-like growth factors, and uptake of intracellular pathogens, being associated with exceptional longevity as well [71] (Additional file 1: Supplementary Results). Accordingly, both nutritional and pathogen-related selective pressures might have triggered such adaptive events, which have been observed so far only at the single-gene level for *FOXO3* [94].

## Conclusions

By taking advantage from high-coverage WGS data, the present study has had the opportunity to infer the demographic and adaptive history of the ancestors of modern Italians with an unprecedented level of resolution. In particular, we provided new evidence for early differentiation dating back to the Late Glacial between population clusters that represent the edges of the cline of Italian variation, as well as for Neolithic and distinct (i.e., steppe-related versus Anatolian/Mediterranean) Bronze Age demographic processes having then continued to differentially shape the gene pool of groups distributed along the peninsula. Moreover, we proposed climate-related selective pressures as potential factors having influenced adaptive evolution at insulin-related genes especially in the ancestors of Northern Italians. By regulating glucose homeostasis, adiposity, and thermogenesis in response to high-calorie diets adopted to cope with energetically demanding environmental conditions, these adaptive events might have also contributed to make people from Northern Italy less prone to develop T2D and obesity despite the challenging nutritional context imposed by modern lifestyles. Conversely, possible adaptations against pathogens and modulation of melanogenesis in response to high UV radiation are supposed to have played a role in reduced susceptibility of people from Southern Italy respectively to immunoglobulin-A nephropathy and skin cancers. Finally, multiple adaptive processes evolved by the overall Italian population, but having resulted more pronounced in people from the southern regions of the peninsula, were found to have the potential to secondarily modulate the longevity phenotype. Therefore, by pinpointing genetic determinants underlying biological adaptation of Italian population clusters in response to locally diverging environmental contexts, the present study succeeded in disclosing also valuable biomedical implications of such evolutionary events. Coupled with the identification of the demographic processes having predominantly shaped the present-day heterogeneous Italian genomic background, this supports once again the usefulness of an evolutionary approach in the dissection of the deep causes of human populations’ health and disease, and highlighted important dynamics that contributed to the formation of the Continental and Southern European genomic landscapes.

## Methods

### Sequenced samples and data curation

A total of 38 unrelated individuals, three generations native (i.e., with all grandparents originating from the same geographical area) from different Italian regions (i.e., Piedmont, Lombardy, Veneto, Emilia-Romagna, Apulia, Calabria, Sicily), were selected among the healthy controls sequenced for the whole genome within the framework of a biomedical study and in order to be the representative of the previously described Northern and Southern Italian population clusters [16]. High-coverage (90×) WGS data were generated by preparing sequencing libraries with the TruSeq DNA PCR-Free Library Preparation Kit (Illumina San Diego, CA, USA) using a 350-bp setting and following the manufacturer’s instructions. The HiSeq X Ten Reagent Kit v2.5 for 2 × 150 cycles and a HiSeq X Ten platform (Illumina San Diego, CA, USA) were then used to carry out sequencing experiments. The obtained sequence reads were aligned against the human reference sequence hg19 (GRCh37) with the Isaac aligner (version 01.14.02.18) by considering a minimum PHRED quality score threshold of 20 from the 3′-end. They were then processed by means of the Isaac Variant Caller (version 1.0.7) tool using default parameters to call and filter high-quality genotypes according to a framework that implements several steps, such as noise filtration based on sequencing and alignment metrics, read realignment, filtration of base calls on the base of mismatch density, heuristic adjustment of same-strand base call quality to reflect potential error dependencies between calls, and calculation of genotype probabilities via a Bayesian model [95]. This pipeline of analyses was chosen because it has been demonstrated to be four to five times faster than traditional approaches (e.g., those based on the GATK tool) but showing comparable accuracy and sensitivity [95]. The initial set of variants detected in the 38 examined individuals was further reduced to 20,075,710 SNVs by removing variants located in tandem repeats and homopolymer regions, as well as those showing a call rate lower than 98% [96]. A transition/transversion (Ts/Tv) ratio of 2.071 was finally calculated, resulting within the 2.0–2.1 range expected for genome-wide datasets [97] and thus attesting the accuracy of the implemented variant calling procedure.

The obtained genotypes were then submitted to the following QC procedures using functions implemented in the PLINK package. In particular, we filtered out SNVs showing more than 5% of missing data and/or characterized by significant deviations from the Hardy-Weinberg equilibrium after Bonferroni correction for multiple testing (*p* < 5.2 × 10^−10^). Moreover, we considered only autosomal variants by removing SNVs located on sex chromosomes and mitochondrial DNA, and we discarded possible ambiguous SNVs (i.e., characterized by A/T or C/G substitutions) when merging our dataset with already published data. According to these QCs, we created a “high-quality Italian dataset” including genotypes for 17,495,290 SNVs from all the sequenced samples.

We next merged these WGS data with an Italian reference dataset made up of genome-wide genotypes from 737 samples with known micro-geographical origins (i.e., at the level of single administrative provinces) and representative of the overall population distributed along the Italian Peninsula [16]. This enabled us to create a “low-density Italian dataset” including 251,648 SNVs, which was submitted to the Procrustes analysis [98] to explore the distribution of the sequenced N_ITA and S_ITA population samples within the well-known north-to-south cline of Italian genetic variation and to check for possible mismatches between their genomic and geographic ancestry. For this purpose, the *smartpca* method implemented in the EIGENSOFT package v6.0.1 [99] was used to perform PCA, and individuals’ coordinates for the most informative PCs were averaged within the sampling provinces and projected from the PCA space onto their geographic coordinates by using the R *vegan* package. In order to implement PCA, the merged “low-density Italian dataset” was further processed to pinpoint potential genetic relatedness among subjects and to filter for variants in high linkage disequilibrium (LD) with each other. In more detail, identity by descent (IBD) estimates were calculated for each pair of subjects as the genome-wide proportion of shared alleles, and only individuals with an IBD kinship coefficient lower than 0.125 were considered. LD pruning was also performed by removing a SNV for each pair showing *r*^2^ > 0.2 within windows of 50 SNVs and advancing by five SNVs.

We also merged our “high-quality Italian dataset” with data generated with the same Illumina sequencing technology for 69 individuals belonging to 35 European and Mediterranean populations [77] to obtain a “high-density Euro-Mediterranean dataset” including 6,993,871 SNVs that was used for haplotype-based population structure and aDNA-guided analyses. For the former purpose, the dataset was phased to infer haplotypes with SHAPEIT2 v2.r790 [100] by using default parameters, HapMap phase 3 recombination maps, and WGS data generated by the 1000 Genomes Project [101] as a reference panel. In order to perform analyses including aDNA samples, the “high-density Euro-Mediterranean dataset” was further pruned and merged with genome-wide genotypes for a panel of 559 ancient samples assembled from literature [1, 3, 4, 6–8, 102]. This led to the creation of a “modern + aDNA dataset” including 47,806 SNVs.

The “high-quality Italian dataset” was finally phased with SHAPEIT2 v2.r790 according to the same approach described for the “high-density Euro-Mediterranean dataset” but using a reconstructed reference human genome sequence as a guide for distinguishing between ancestral and derived alleles. Ancestral/derived state of each allele in such a reference sequence was previously assigned by aligning it with the Ensembl Compara 6 primates EPO genome sequences [103]. In particular, only alleles conserved in all the compared genomes were considered as ancestral. A “phased high-quality Italian dataset” including 13,381,038 SNVs with known ancestral/derived states was thus obtained and used for selection scans.

### Haplotype sharing clustering analyses

To formally test whether the generated WGSs were representative of distinct genetically homogenous Italian population clusters, we applied the haplotype-based methods implemented in the CHROMOPAINTER/fineSTRUCTURE pipeline [104] to the phased “high-density Euro-Mediterranean dataset”. CHROMOPAINTERv2 was run to reconstruct patterns of haplotype sharing of each individual by using all the other samples included in the dataset as potential “donors” but excluding themselves (i.e., preventing self-copy). We thus estimated the mutation/emission and recombination/switch rates using 10 steps of the expectation-maximization algorithm on a subset of chromosomes {4,10,15,22}. The mean values calculated across all autosomes/individuals and weighted by the number of SNVs were then used to run the final CHROMOPAINTER analysis on all chromosomes by using *k* = 100 as the number of expected haplotype chunks to define a genomic region. The obtained matrix of counts of shared haplotype chunks across all autosomes was then used as input for fineSTRUCTURE version fs2.1 [104]. We ran the algorithm with 1,000,000 “burn-in” iterations of MCMC, followed by another 1,000,000 iterations and sampling the inferred clustering patterns every 10,000 runs. We then performed 100,000 additional hill-climbing steps to improve posterior probability and to merge the identified clusters in a step-wise fashion. The described population clusters were finally defined by collapsing branches of the obtained fineSTRUCTURE dendrogram up to the second last splitting point to reduce the number of small, closely related and scarcely supported clusters. Moreover, also some clades observable at a higher level of the fineSTRUCTURE tree, but splitting with a posterior probability lower than 80%, were collapsed until reaching the subsequent branching point showing a posterior probability above such a threshold.

### Inferring and dating recent admixture events

The GLOBETROTTER pipeline [105] was applied to the phased “high-density Euro-Mediterranean dataset” to fine map and date relatively recent admixture events involving the 12 Italian and Euro-Mediterranean population clusters identified with fineSTRUCTURE.

Differently from the CHROMOPAINTER run previously described as concerns the clustering analysis, here, the total length of haplotype chunks for each recipient individual and copied from every other donor was averaged over all samples belonging to a given cluster. Moreover, to infer the potentially different mixing proportions of N_ITA e S_ITA with respect to the other population groups, we considered all the individuals as recipients, but we excluded the two Italian clusters from the donors. The obtained matrix was then submitted to the GLOBETROTTER pipeline. Accordingly, we first inferred N_ITA and S_ITA mixture proportions with the *nnls* function, as previously described [105]. Then, we performed the dating procedure by following recommendations indicated in [106] and by running computations first with the null individual option. For this purpose, we tested in turn each possible pair of parental groups chosen from the 10 Euro-Mediterranean population clusters by performing a first run to infer admixture proportions, dates (assuming 29 years per generation [107]), and sources of admixture. A second run was then performed according to these results and by implementing 100 times bootstrap resampling to infer confidence intervals around the obtained estimates.

### Exploring relationships between modern and ancient populations

The “modern + aDNA dataset” was used to formally test for differential genetic relationships of present-day Italian population clusters identified by the fineSTRUCTURE analysis with a large panel of ancient Eurasian samples. For this purpose, PCA was first performed by using the *smartpca* method implemented in the EIGENSOFT package v6.0.1 [99] and by applying the *lsqproject* option to overcome issues related to the potential high rate of missing genotypes in aDNA data. Then, we computed outgroup *f3* statistics in the form of *f3* (CHB; X Italian population cluster, X ancient population cluster) by using the ADMIXTOOLS *qp3pop* function [108] and by grouping ancient samples according to their archeological/temporal frameworks and to the genetic clustering pointed out by PCA. We finally contrasted the two present-day Italian population clusters according to their levels of shared genetic drift with each ancient population group. In particular, differences (i.e., residuals) in their outgroup *f3* scores were calculated and those exceeding ± 2 SDs from the mean of the obtained distribution were considered as significant.

### Estimates of effective population sizes and split times

The SMC++ method [109] was used to explicitly model the demographic histories of Italian population clusters and to compare them with that of CEU by taking advantage of both LD information provided by the coalescent hidden Markov model and information derived from the sample frequency spectrum. This enabled us to estimate the changes in *N*_e_ over time for each group, as well as their genetic split times. For this purpose, we set 150 generations as the most recent time point for population size inference (T1) and 10 spline knots to anchor the size history according to ad hoc simulations of a model of population growth for people of European ancestry [27]. The obtained scaled estimates of *N*_e_ and split times were then converted into real estimates by considering a mutation rate of 1.25 × 10^−8^ mutations per nucleotide per generation [27] and a generation time of 29 years [107].

### Detecting genomic signatures of natural selection

The “phased high-quality Italian dataset” was used to infer adaptive evolution of the Italian population clusters identified by the fineSTRUCTURE analysis. Two independent and complementary statistics, such the DIND and nSL, were computed to detect different typologies of selective events due to positive selection. In particular, with respect to other haplotype-based tests, the DIND statistics provided robustness to variation in sequencing coverage and low sample sizes [110], while the nSL enabled to properly account for variation in recombination rates and for the confounding effects due to demography [111]. The BALLET pipeline [112] was further applied to test for the occurrence of events of balancing selection. For these purposes, we first filtered out SNVs showing derived allele frequency lower than 0.2, as they were proved to bias DIND results [113], and we calculated DIND scores for each variant by using self-customized Python scripts. The *selscan* v1.1.0b package [114] was instead used to compute nSL scores for each SNV by considering windows of maximum 4500 consecutive loci. A composite likelihood method requiring information about an outgroup species (i.e., *P. troglodytes*) was then used to calculate the probability of each nucleotide site to be polymorphic under a model of balancing selection. SNVs presenting only the ancestral allele (i.e., showing no differences between the tested and outgroup species) were removed, and the number of within-species polymorphisms and between-species substitutions for each site was calculated. Such estimates, along with whole-genome recombination maps and a coalescent time between humans and chimpanzees of six million years ago, were used as input for the BALLET pipeline. Finally, in order to shortlist selection signatures specific of the Italian groups, which are thus plausibly ascribable to a combination of nature, duration, and intensity of selective pressures that was peculiar of the Italian Peninsula, the analyses mentioned above were replicated on CEU, and IBS WGS data generated by the 1000 Genomes Project [101] and signals shared between Italians and the other populations of Western European ancestry were filtered out.

### Gene network analyses

Combinations of favorable variants in multiple adaptive haplotypes at moderate frequency, rather than remarkable increase in frequency of a single haplotype (as supposed under the *hard sweep* model), represent the main genomic footprint of polygenic adaptation [37, 38]. Therefore, traditional single gene-oriented selection scans showed limited power in detecting this typology of selective signatures, and data not affected by ascertainment bias towards common SNPs are essential to detect them. To test a model as close as possible to that of polygenic adaptation, instead of considering SNV/gene-level results from the abovementioned selection scans, we used the *signet* algorithm implemented in the dedicated R package [115] to analyze the obtained genome-wide distributions of DIND, nSL, and BALLET scores to identify gene networks enriched for weak but pervasive signatures of natural selection. This approach enabled us to explore the possibility that natural selection acted at a functional pathway as a whole or, more likely, at circumscribed gene subnetworks involved in a given biological function, rather than on single genes [38]. Information about the gene/genes located up to 50 kb upstream and downstream of each tested SNV was retrieved, and the highest DIND, nSL, and BALLET scores within such a range were considered as representatives of the gene of interest. Functional pathways related to these input genes were reconstructed according to the Kyoto Encyclopedia of Genes and Genomes (KEGG) database and used to test for significant shifts towards extreme *signet* values in the distribution of scores observed within annotated pathways as previously detailed [38]. Significant gene subnetworks (*p* < 0.05) were thus identified for each population cluster and according to each of the computed selection statistics, being finally plotted using Cytoscape v3.6.0 [116].

## Supplementary information

**Additional file 1 **: **Figure S1.** Procrustes analysis projecting genomic information summarized by first and second principal components onto geographic coordinates of Italian population samples. **Figure S2.** Decay of the length of chromosome chunks inherited by Italian population clusters from possible pairs of parental groups calculated with the GLOBETROTTER pipeline. **Figure S3.** PCA projecting variation of 559 ancient samples onto the genetic space defined by 239 individuals belonging to 40 modern Euro-Mediterranean populations. **Figure S4.** Outgroup *f3* biplot comparing shared genetic drift between the N_ITA and S_ITA population clusters and, in turn, all ancient population groups included in the “modern + aDNA dataset”. **Figure S5.** Representation of the *Insulin secretion* pathway and of its components subjected to positive selection in the Italian population. **Figure S6.** Representation of the *Mucin type O-glycan biosynthesis* pathway and of its components subjected to positive selection in the S_ITA cluster. **Figure S7.** Representation of the *Basal cell carcinoma* pathway and of its components subjected to positive selection in the S_ITA cluster. **Table S1.** Admixture proportions inferred for N_ITA and S_ITA population clusters with the GLOBETROTTER method. **Table S2.** Admixture dates inferred for N_ITA and S_ITA population clusters with the GLOBETROTTER method. **Table S3.** Gene networks showing significant signatures of positive selection according to *signet* analysis performed on the obtained genome-wide distribution of DIND scores. **Table S4.** Gene networks showing significant signatures of positive selection according to *signet* analysis performed on the obtained genome-wide distribution of nSL scores. **Table S5.** Gene networks showing significant signatures of balancing selection according to signet analysis performed on the obtained genome-wide distribution of BALLET scores. **Supplementary Results.**

## Data Availability

All data generated or analyzed during this study are included in this published article, its supplementary information files, and publicly available repositories. In particular, data generated during this study have been deposited at the figshare repository under the item “Italian dataset” [117]; whole-genome sequence data for European and Mediterranean populations were downloaded from the EBI European Nucleotide Archive and the 1000 Genomes Project database under accession numbers PRJEB9586, ERP010710, and estd219 [118, 119], while genome-wide data for ancient samples were retrieved from the EBI European Nucleotide Archive under accession numbers PRJEB11450, PRJEB13123, and PRJEB14455 [120–122].

## Abbreviations

LGM: Last Glacial Maximum
SNPs: Single nucleotide polymorphisms
WGS: Whole-genome sequence
N_ITA: Northern Italian population cluster
S_ITA: Southern Italian population cluster
QC: Quality control
SNVs: Single nucleotide variants
aDNA: Ancient DNA
IBS: People from the Iberian Peninsula
PCA: Principal component analysis
SMC++: Sequential Markov coalescent + plenty of unlabeled samples
Ne: Effective population size
CEU: People of Northern and Western European ancestry
DIND: Derived intra-allelic nucleotide diversity
nSL: Number of segregating sites by length
BALLET: Balancing selection likelihood test
ADCY: Adenylate cyclase
T2D: Type II diabetes
FZD: Frizzled G protein-coupled receptors
MAPK: Mitogen-activated protein kinase
ALDH: Aldehyde dehydrogenase
IgAN: Immunoglobulin-A nephropathy
UV: Ultraviolet
IBD: Identity by descent
LD: Linkage disequilibrium
KEGG: Kyoto Encyclopedia of Genes and Genomes
CHB: Han Chinese
SD: Standard deviation
