# Supplementary material for: Genomic history of the Italian population recapitulates key evolutionary dynamics of both Continental and Southern Europeans
Source: BMC Biol. 2020 May 22;18:51. doi: 10.1186/s12915-020-00778-4 (PMC7243322; doi:10.1186/s12915-020-00778-4)
Supplement: Supplementary file 1 — Additional file 1 : Figure S1. Procrustes analysis projecting genomic information summarized by first and second principal components onto geographic coordinates of Italian population samples. Figure S2. Decay of the length of chromosome chunks inherited by Italian population clusters from possible pairs of parental groups calculated with the GLOBETROTTER pipeline. Figure S3. PCA projecting variation of 559 ancient samples onto the genetic space defined by 239 individuals belonging to 40 modern Euro-Mediterranean populations. Figure S4. Outgroup f3 biplot comparing shared genetic drift between the N_ITA and S_ITA population clusters and, in turn, all ancient population groups included in the “modern + aDNA dataset”. Figure S5. Representation of the Insulin secretion pathway and of its components subjected to positive selection in the Italian population. Figure S6. Representation of the Mucin type O-glycan biosynthesis pathway and of its components subjected to positive selection in the S_ITA cluster. Figure S7. Representation of the Basal cell carcinoma pathway and of its components subjected to positive selection in the S_ITA cluster. Table S1. Admixture proportions inferred for N_ITA and S_ITA population clusters with the GLOBETROTTER method. Table S2. Admixture dates inferred for N_ITA and S_ITA population clusters with the GLOBETROTTER method. Table S3. Gene networks showing significant signatures of positive selection according to signet analysis performed on the obtained genome-wide distribution of DIND scores. Table S4. Gene networks showing significant signatures of positive selection according to signet analysis performed on the obtained genome-wide distribution of nSL scores. Table S5. Gene networks showing significant signatures of balancing selection according to signet analysis performed on the obtained genome-wide distribution of BALLET scores. Supplementary Results. [file 12915_2020_778_MOESM1_ESM.docx]

**Genomic history of the Italian population recapitulates key evolutionary dynamics of both Continental and Southern Europeans**

Marco Sazzini^#,†^, Paolo Abondio^#^, Stefania Sarno^#^, Guido Alberto Gnecchi-Ruscone^#^, Matteo Ragno, Cristina Giuliani, Sara De Fanti, Claudia Ojeda-Granados, Alessio Boattini, Julien Marquis, Armand Valsesia, Jerome Carayol, Frederic Raymond, Chiara Pirazzini, Elena Marasco, Alberto Ferrarini, Luciano Xumerle, Sebastiano Collino, Daniela Mari, Beatrice Arosio, Daniela Monti, Giuseppe Passarino, Patrizia D'Aquila, Davide Pettener, Donata Luiselli, Gastone Castellani, Massimo Delledonne, Patrick Descombes, Claudio Franceschi^*^, Paolo Garagnani^*,†^

^#^These authors contributed equally to this work. *These authors contributed equally to this work.

^†^Corresponding authors: E-mail: marco.sazzini2@unibo.it; paolo.garagnani2@unibo.it

**Additional files**

**Additional file 1: Figure S1.** Procrustes analysis projecting genomic information summarized by first and second principal components onto geographic coordinates of Italian population samples. **Figure S2.** Decay of the length of chromosome chunks inherited by Italian population clusters from possible pairs of parental groups calculated with the GLOBETROTTER pipeline. **Figure S3.** PCA projecting variation of 559 ancient samples onto the genetic space defined by 239 individuals belonging to 40 modern Euro-Mediterranean populations. **Figure S4.** Outgroup *f3* biplot comparing shared genetic drift between the N_ITA and S_ITA population clusters and, in turn, all ancient population groups included in the “modern + aDNA dataset”. **Figure S5.** Representation of the *Insulin secretion* pathway and of its components subjected to positive selection in the Italian population. **Figure S6.** Representation of the *Mucin type O-glycan biosynthesis* pathway and of its components subjected to positive selection in the S_ITA cluster. **Figure S7.** Representation of the *Basal cell carcinoma* pathway and of its components subjected to positive selection in the S_ITA cluster. **Table S1.** Admixture proportions inferred for N_ITA and S_ITA population clusters with the GLOBETROTTER method. **Table S2.** Admixture dates inferred for N_ITA and S_ITA population clusters with the GLOBETROTTER method. **Table S3.** Gene networks showing significant signatures of positive selection according to *signet* analysis performed on the obtained genome-wide distribution of DIND scores. **Table S4.** Gene networks showing significant signatures of positive selection according to *signet* analysis performed on the obtained genome-wide distribution of nSL scores. **Table S5.** Gene networks showing significant signatures of balancing selection according to signet analysis performed on the obtained genome-wide distribution of BALLET scores. **Supplementary Results.**


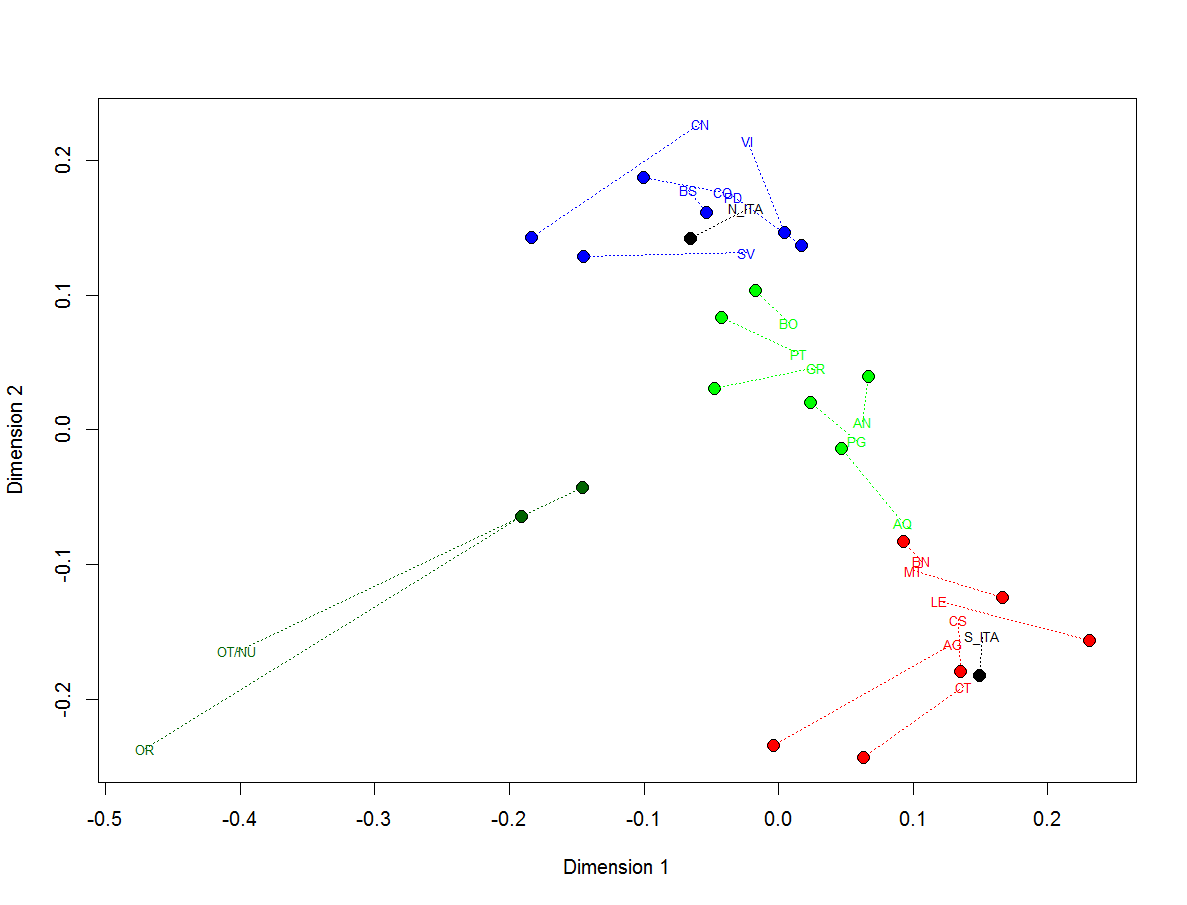


**Figure S1.** Procrustes analysis projecting genomic information summarized by first and second principal components onto geographic coordinates of Italian population samples. Genomic information resulting from PCA applied to the “low-density Italian dataset” (and from averaging individuals’ coordinates related to the most informative PCs within sampling locations) is represented by codes of population samples, while geographic coordinates are displayed by colored dots. Colors differentiate population clusters suggested by geographic coordinates, while dotted lines visualize residuals of genetic/geographic regression. Italian provinces belonging to the Northern Italian, Central Italian, Southern Italian and Sardinian clusters are displayed in blue, green, red, and dark green, respectively, and were previously described in [16]. Codes and geographic coordinates of population samples sequenced for the whole genome in the present study are instead displayed in black.


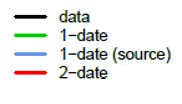


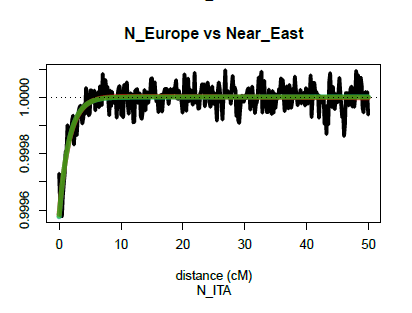

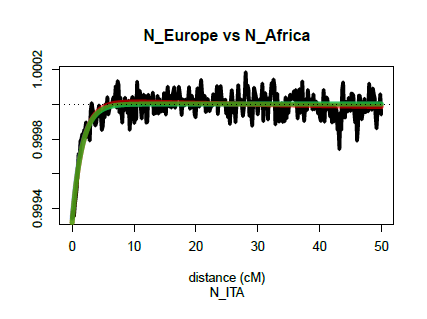

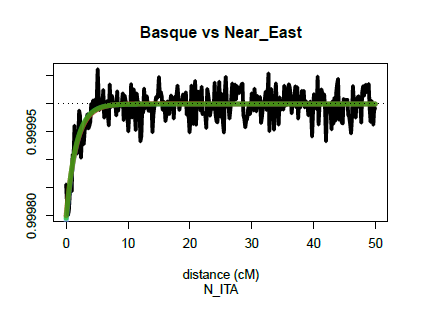

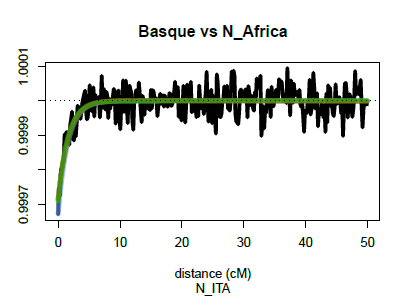


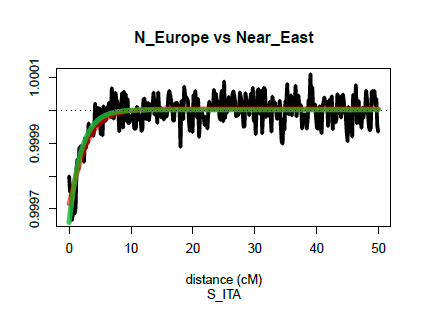

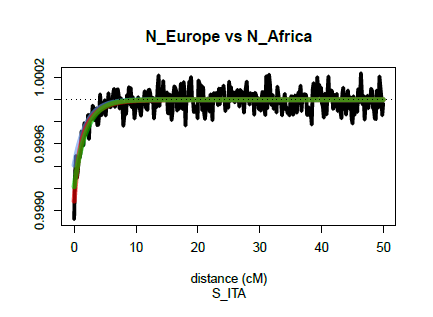


**Figure S2.** Decay of the length of chromosome chunks inherited by Italian population clusters from possible pairs of parental groups calculated with the GLOBETROTTER pipeline. Interpolations of the inherited genome segments length decay curve supporting Northern European populations (and Basque in the case of N_ITA) and Near Eastern/North African groups as source of gene flow having affected the examined Italian population clusters.

**
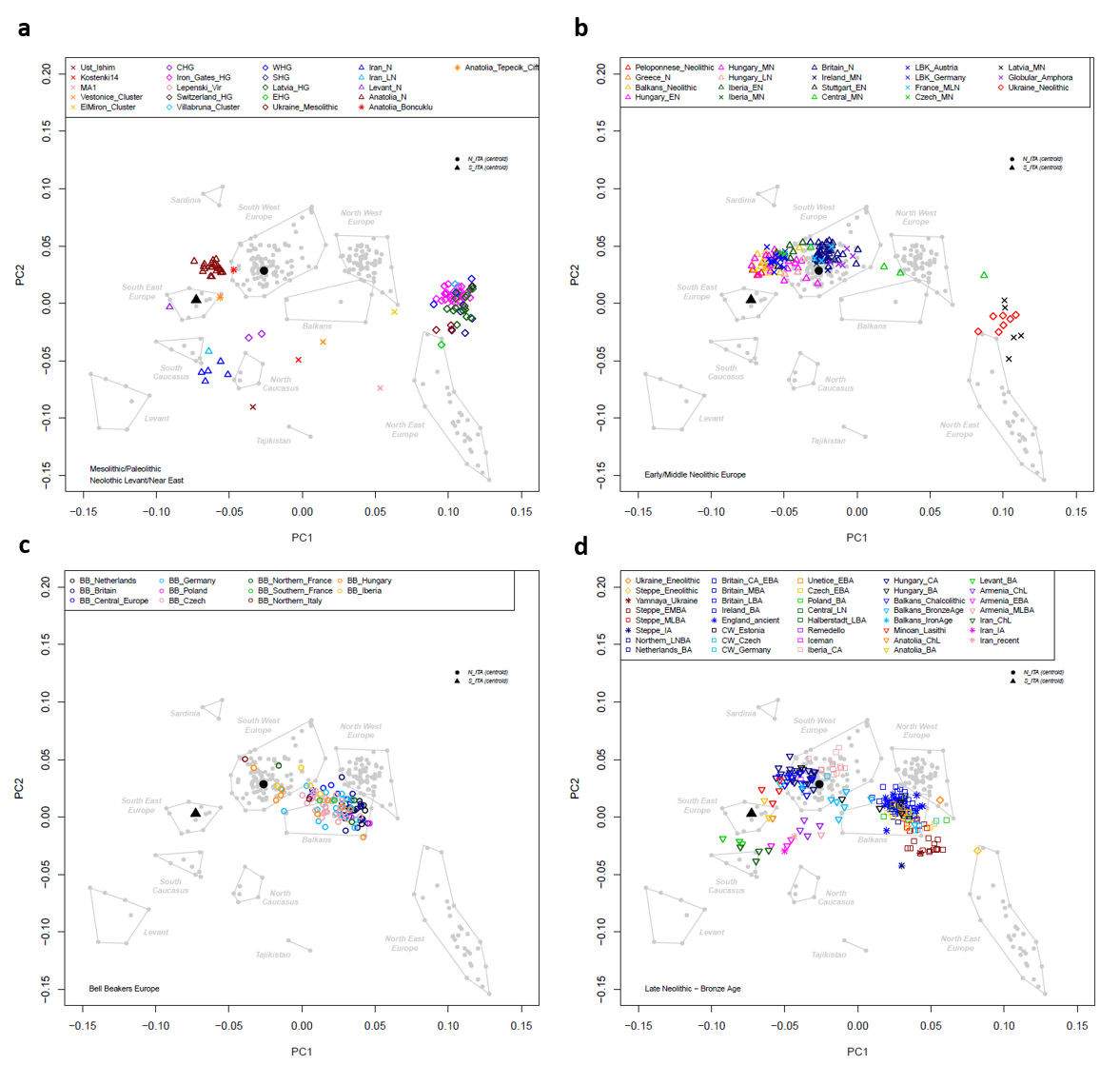
**

**Figure S3.** PCA projecting variation of 559 ancient samples onto the genetic space defined by 239 individuals belonging to 40 modern Euro-Mediterranean populations. Modern samples are reported as grey dots, with the exception of centroids calculated for the N_ITA e S_ITA clusters (black dot and black triangle, respectively). The main ancient groups identified according to their temporal, geographical or cultural framework are highlighted progressively in the different plots as follows: **a** Paleolithic/Mesolithic and Levant/Near East Neolithic; **b** European Early/Middle Neolithic; **c** European Bell Baker; **d** Late Neolithic and Bronze Age.


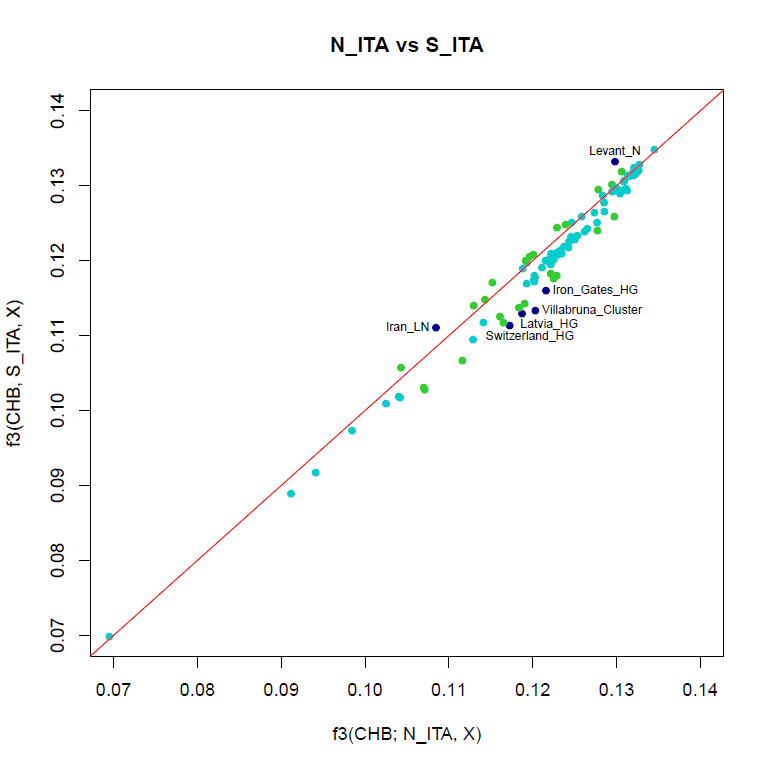


**Figure S4.** Outgroup *f3* biplot comparing shared genetic drift between the N_ITA and S_ITA population clusters and, in turn, all ancient population groups included in the “modern + aDNA dataset”. Each axis reports scores for the outgroup *f3* statistics in the form of *f3*(CHB; Italian cluster, X ancient population). Scores computed for N_ITA are reported on the x-axis, while those obtained for S_ITA are reported on the y-axis. The red line represents the x = y straight line. The closer the points are to the red line the more the two tested Italian clusters overlap in their shared genetic ancestry with respect to the corresponding ancient population. Differences in outgroup *f3* scores exceeding ± 2 SDs from the mean of the distribution were considered significant and marked in blue, while differences lying over one SD were marked in green (see legend of Fig. 2 for more details).

**
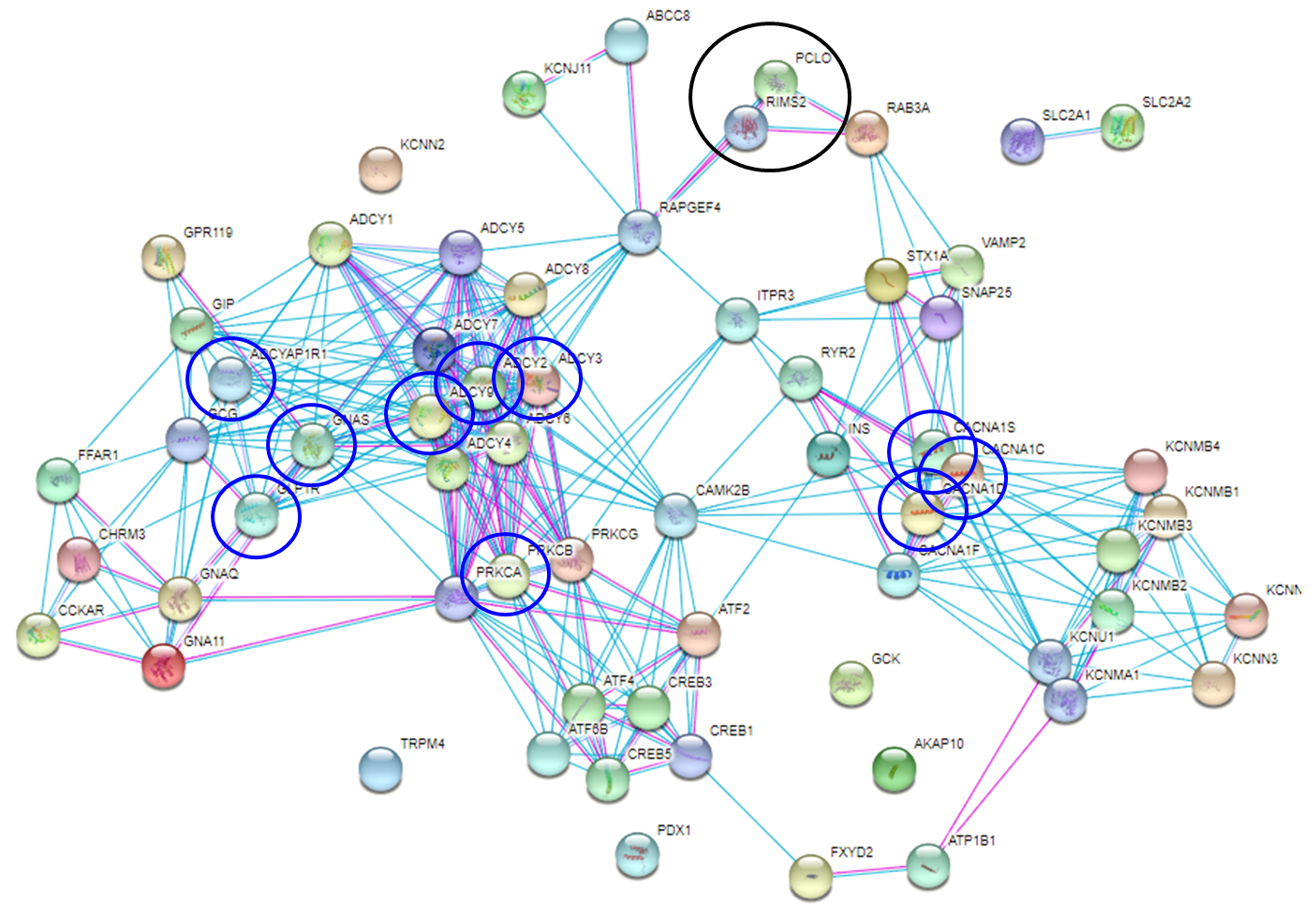
**

**Figure S5.** Representation of the *Insulin secretion* pathway and of its components subjected to positive selection in the Italian population. The *Insulin secretion* pathway (ko04911) was reconstructed according to the gene list implemented in the Kyoto Encyclopedia of Genes and Genomes (KEGG) [39] and known interactions among loci belonging to it were displayed using STRING [40]. Interactions supported by experimental data are highlighted by pink lines, while those inferred from database information are represented by light blue lines. The black circle includes genes subjected to positive selection in both N_ITA and S_ITA clusters, whereas blue circles point to genes having undergone adaptive evolution only in N_ITA. Most of them turned out to belong to the *Thermogenesis* (ko04714), *Regulation of lipolysis in adipocytes* (ko04923), *Type II diabetes mellitus* (ko04930), *Glucagon signaling* (ko04922), and *Longevity regulating* (ko04211) pathways as well.

**
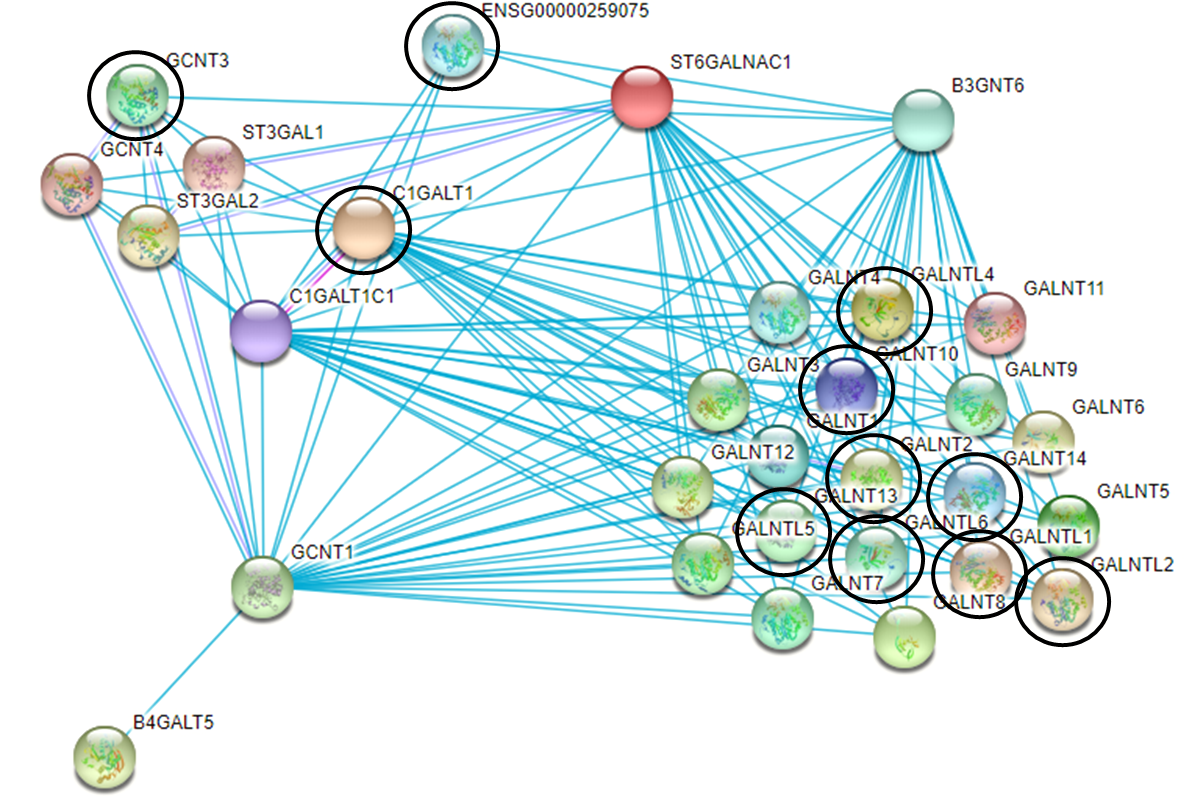
**

**Figure S6.** Representation of the *Mucin type O-glycan biosynthesis* pathway and of its components subjected to positive selection in the S_ITA cluster. The *Mucin type O-glycan biosynthesis* pathway (ko00512) was reconstructed according to the gene list implemented in the KEGG database [39] and known interactions among loci belonging to it were displayed using STRING [40]. Interactions supported by experimental data are highlighted by pink lines, while those inferred from database information are represented by light blue lines. Black circles point to genes subjected to positive selection in S_ITA.

**
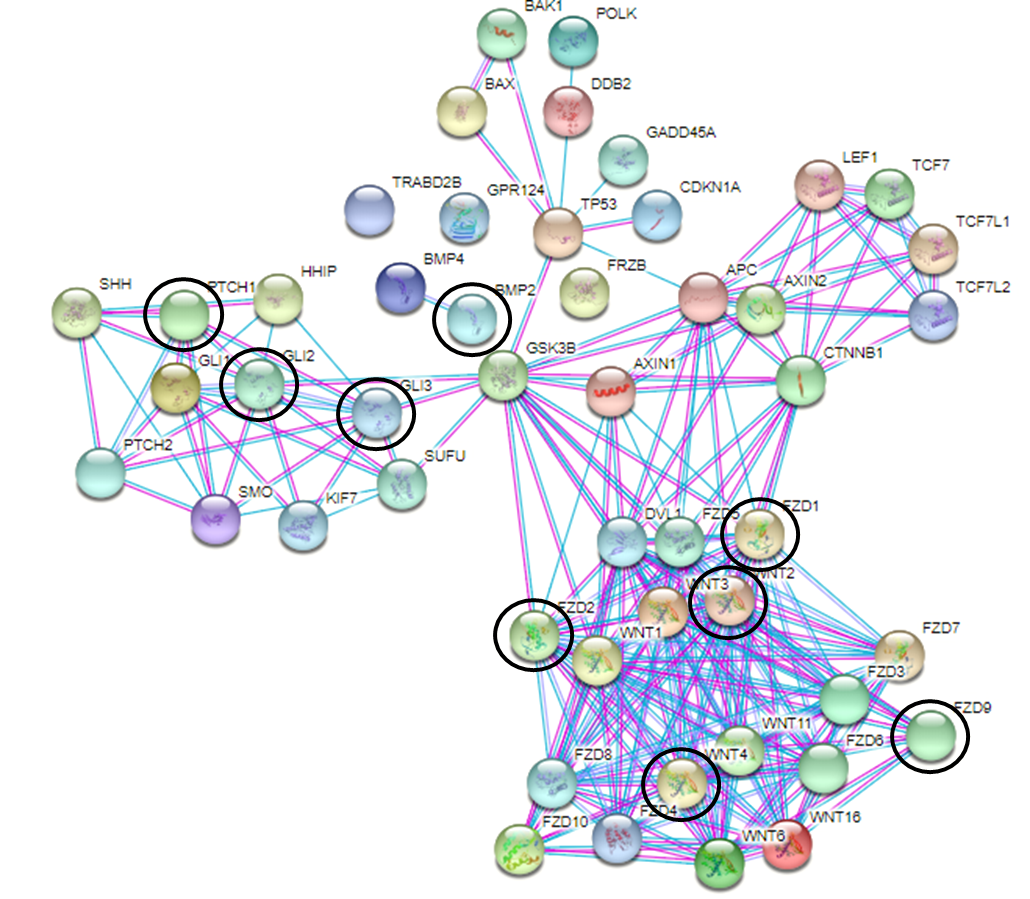
**

**Figure S7.** Representation of the *Basal cell carcinoma* pathway and of its components subjected to positive selection in the S_ITA cluster. The *Basal cell carcinoma* pathway (ko05217) was reconstructed according to the gene list implemented in the KEGG database [39] and known interactions among loci belonging to it were displayed using STRING [40]. Interactions supported by experimental data are highlighted by pink lines, while those inferred from database information are represented by light blue lines. Black circles point to genes subjected to positive selection in S_ITA. Most of them turned out to belong to the *Melanogenesis* (ko04916) and *mTOR signaling* (ko04150) pathways as well.

**Table S1.** Admixture proportions inferred for N_ITA and S_ITA population clusters with the GLOBETROTTER method.

| Cluster | Admixture  source | Admixture proportion | Relative contributions | | |
| --- | --- | --- | --- | --- | --- |
| N_ITA | Major | 59% | N_Europe (79%) | Basque (21%) |  |
|  | Minor | 41% | Near East (78%) | N_Africa (19%) | Beduin (2.4%) |
| S_ITA | Major | 68% | Near East (91%) | Beduin (7%) | N_Africa (2%) |
|  | Minor | 32% | N_Europe (100%) |  |  |

Relative contributions, % of different admixture components contributing to the major

or minor admixture proportion.

**Table S2.** Admixture dates inferred for N_ITA and S_ITA population clusters with the GLOBETROTTER method.

| Cluster | Date  (N gen.) | 95% CI (N gen.) | Date (ya) | 95% CI (ya) |
| --- | --- | --- | --- | --- |
| N_ITA | 62 | 51.7 - 71.5 | 1,798 | 1,499 - 2,074 |
| S_ITA | 50 | 41.9 - 63.1 | 1,450 | 1,215 - 1,829 |

N gen., number of generations; CI, confidence interval; ya, years ago.

**Table S3.** Gene networks showing significant signatures of positive selection according to *signet* analysis performed on the obtained genome-wide distribution of DIND scores.

| Cluster | Pathway | Pathway  size | Network  size | HSS | p-value | Genes |
| --- | --- | --- | --- | --- | --- | --- |
| N_ITA | Insulin secretion | 50 | 2 | 7.238 | 0.034 | *RIMS2 PCLO* |
| S_ITA | Insulin secretion | 50 | 2 | 7.972 | 0.032 | *RIMS2 PCLO* |
|  | Mucin type O-glycan biosynthesis | 29 | 5 | 8.386 | 0.023 | *GALNT10 C1GALT1 GALNT13 GALNTL6 ENSG00000259075* |

Pathway size, number of genes belonging to the pathway; Network size, number of genes composing the identified network; HSS, highest scoring subnetwork; p-value, rank p-value calculated by comparing observed HSS with a null distribution of HSS generated for each subnetwork of a specific size via 20,000 permutations.

**Table S4.** Gene networks showing significant signatures of positive selection according to *signet* analysis performed on the obtained genome-wide distribution of nSL scores.

| Cluster | Pathway | Pathway  size | Network  size | HSS | p-value | Genes |
| --- | --- | --- | --- | --- | --- | --- |
| N_ITA | Insulin secretion | 50 | 10 | 6.556 | 0.016 | *ADCY2 ADCY3 ADCY9 ADCYAP1R1 CACNA1C CACNA1D CACNA1S GLP1R GNAS PRKACA* |
| S_ITA | Mucin type O-glycan biosynthesis | 29 | 11 | 5.573 | 0.034 | *GALNT2 GCNT3 GALNT10 C1GALT1 GALNTL1 GALNTL6 GALNT14 GALNT13 GALNTL2 GALNTL4* |
|  | Basal cell carcinoma | 45 | 13 | 5.542 | 0.035 | *BMP2 FZD2 GLI2 GLI3 PTCH1 WNT7A WNT7B WNT8A WNT2B FZD1 FZD10 WNT4 WNT5B* |

Pathway size, number of genes belonging to the pathway; Network size, number of genes composing the identified network; HSS, highest scoring subnetwork; p-value, rank p-value calculated by comparing observed HSS with a null distribution of HSS generated for each subnetwork of a specific size via 20,000 permutations.

**Table S5.** Gene networks showing significant signatures of balancing selection according to signet analysis performed on the obtained genome-wide distribution of BALLET scores.

| Cluster | Pathway | Pathway  size | Network  size | HSS | p-value | Genes |
| --- | --- | --- | --- | --- | --- | --- |
| N_ITA | FoxO signaling | 59 | 16 | 4.849 | 0.017 | *ADCY2 ADCY5 ADCY8 ADCY9 CACNA1A CACNA1B CNR1 GNG10 KCNJ6 PRKACG MAPK1 MAPK11 MAPK10 MAPK12 GNG13 GNG2* |
|  | Glycerolipid metabolism | 87 | 12 | 4.648 | 0.024 | *DGKB PLA2G1B PLD1 PLA2G6 PLPP1 DGKI CEPT1 PEMT PISD PLA2G3 LPCAT1 PLB1* |
|  | Glycolysis/ gluconeogenesis | 30 | 10 | 4.764 | 0.019 | *ALDH2 ALDH3A1 ALDH1A3 ALDH3B2 ALDH3A2 AOC2 GAD2 AOC3 UPB1 CARNS1* |
|  | Glucagon signaling/insulin resistance | 88 | 7 | 4.367 | 0.039 | *ITPR1 PLCG2 PRKCE PRKCG PRKCH PLCB1 TRPV4* |
| S_ITA | FoxO signaling | 59 | 20 | 4.242 | 0.031 | *ADCY1 ADCY2 ADCY5 ADCY6 ADCY8 ADCY9 CACNA1A CACNA1B CNR1 GNG7 GNGT1 GNGT2 KCNJ3 KCNJ5 KCNJ6 MAPK1 MAPK11 MAPK10 MAPK12 GNG2* |
|  | Glycerolipid metabolism | 87 | 27 | 4.843 | 0.012 | *CDS1 DGKB DGKG PLA2G4A PLD1 PLA2G6 DGKD PLA2G4C PLPP1 PLPP3 CDS2 DGKI LPIN2 CEPT1 PEMT LPIN1 PISD PLA2G3 LPCAT2 AGPAT4 GPAM SELENOI PLD4 PLB1 MBOAT1 PLA2G4F PNPLA7* |
|  | Longevity regulating | 55 | 9 | 4.224 | 0.033 | *GNA12 GNAZ GRM1 PLA2G4A PPP2R1A PRKCG MAPK1 PLA2G4C PLA2G4F* |

Pathway size, number of genes belonging to the pathway; Network size, number of genes composing the identified network; HSS, highest scoring subnetwork; p-value, rank p-value calculated by comparing observed HSS with a null distribution of HSS generated for each subnetwork of a specific size via 20,000 permutations.

**Supplementary Results**

By applying gene network-based analyses aimed at testing for the occurrence of selective events at multiple loci involved in a given biological function, rather than at single genes, we have had the opportunity to investigate for the first time the adaptive evolution of the Italian population according to theoretical assumptions that represent the as realistic as possible proxy for a polygenic adaptation model.

**Signatures of positive selection at insulin-related genes**

Both selection scans performed to test for the occurrence of positive and balancing selection suggested a complex pattern of adaptive evolution for insulin-related genes in the Italian people.

In detail, selective events at *RIMS2* and *PCLO* genes were supposed to have occurred in the common ancestors of N_ITA and S_ITA clusters (Additional file 1: Figure S5, Table S3), with the former locus showing the strongest selection signal and being indicated by network analysis as influencing the activity of the latter. *RIMS2*, which showed the highest DIND score, encodes for a protein highly expressed in pancreatic beta cells where together with the *PCLO* protein product participates to the formation of a Ca^++^ dependent complex responsible for cyclic AMP (cAMP)-induced exocytosis of insulin containing granules [41, 42].

Events of positive selection presumably more recent were instead found to characterize exclusively people from N_ITA, being distributed among ten genes that play a role at different levels of the signaling cascade leading to insulin secretion (Additional file 1: Figure S5, Table S4). Among these loci, the receptor for glucagone-like peptide (GLP-1) mediates not only insulin exocytosis, but exerts also positive influence on insulin gene transcription, mRNA stability and biosynthesis [43], thus regulating key processes that contribute to glucose homeostasis and prevent the development of T2D [42, 44]. Other putative targets of selection (i.e. *CACNA1C* and *CACNA1D*) when mutated were previously reported to be involved in impairment of glucose tolerance eventually leading to T2D as well. The most pervasive signature of selection was however observed at ADCY genes, which catalyze the conversion of ATP into the cAMP that acts as a second messenger participating to several signal transduction pathways, among which those responsible for insulin secretion (as previously discussed for *RIMS2* and *PCLO*), regulation of lipolysis in adipocytes, glucagon signaling and, especially, thermogenesis [45]. In particular, *ADCY2*, *ADCY9,* and *ADCY3* were those characterized by the largest number of connections within the network and *ADCY3* showed the strongest selection signal being also recently confirmed to be involved also in fatty acid oxidation and regulation of adiposity [46, 47]. For instance, homozygous *ADCY3* variants in consanguineous families from Pakistan were found to relate to serious obesity in children [48], while some polymorphisms observed in the Swedish and Chinese populations were associated with decreased risk of such a pathology [49, 50]. Moreover, a series of rare *ADCY3* loss-of-function changes, and especially a variant that decreases its RNA expression and favors the increase of body mass index, were proved to be related to augmented susceptibility to obesity and T2D in the Greenlandic population [51]. In mice subjected to a high-fat diet, an *ADCY3* gain-of-function mutation was also found to determine low basal insulin and glucose levels, as well as reduced risk of obesity [52], while the gene haploinsufficiency is proved to increase disease susceptibility and to diminish expression of genes involved in thermogenesis in subcutaneous adipose tissue [53]. In fact, *ACDY3* upregulation in the brown adipose tissue of rats was observed during the neonatal period when maintenance of body temperature represents a particularly challenging task [54]. In detail, after sensing of a specific extracellular stimulus by accumulation of cAMP, the pathway that regulates thermogenic processes is activated similarly to that modulating insulin secretion. This leads to a signaling cascade culminating in *PGC-1α* activation in adipocytes, which then induces the transcription of downstream thermogenic genes.

**Signatures of positive selection at mucin genes and at loci involved in melanogenesis**

When considering adaptive events specific of S_ITA, genes encoding for mucins and loci participating to melanogenesis emerged as putative targets of positive selection (Fig. 4).

In addition to their physical protection from pathogens at the level of mucosal surfaces, especially in the gastrointestinal tract, mucins are known to play a role in innate immune responses mediated by cationic antimicrobial peptides and to contribute in modulating inflammation [55].

Overall, 12 mucin genes were pointed out as putative targets of selection in S_ITA, four of which (i.e. *C1GALT1*, *GALNTL6*, *GALNT13* and *GALNT10*) being confirmed by both the performed selection scans (Additional file 1: Figure S5, Tables S3-S4). In particular, *C1GALT1,* which encodes for a molecular chaperon indispensable for proper glycosylation of several mucin type O-glycans, represented the central node of both DIND- and nSL-based gene networks, being suggested to influence the action of six mucin genes and to be regulated by the remaining ones. It is expressed especially in kidney, being involved in thrombopoiesis and kidney homeostasis. In fact, *C1GALT1* induced deletion in mice leads to proteinuria rapidly progressing in glomerular sclerosis and kidney fail [56] and several genome-wide association studies reported correlation of some of its variants to Immunoglobulin-A nephropathy (IgAN), which is the most common human kidney inflammation [57]. Interestingly, genetic susceptibility to such a disease was shown to have a peculiar geographical pattern, being widespread in Asia, moderately prevalent in people of European ancestry, and very rare in African populations [58].

Among microorganisms able to inactivate mucins, *Pseudomonas aeruginosa*, the parasitic amoebozoan *Entamoeba histolytica* and the proteobacterium *Burkholderia cepacia* present a geographical distribution correlating negatively to that of IgAN and positively to environmental temperature. They indeed show the highest infection rates in tropical and subtropical regions, showing intermediate diffusion in North Africa and the Near East and being almost absent in Central and Northern Europe. In detail, *Burkholderia cepacia* belongs to a group of Gram-negative proteobacteria that is composed of at least twenty species, which generally cause pneumonia in preterm infants and immunocompromised individuals with underlying lung diseases, such as cystic fibrosis, chronic granulomatous disease and hemoglobinopathies [59]. Worldwide distribution of meiloidosis endemicity (a Burkholderia infection-related pathology), reveals higher frequency in tropical and subtropical regions. More specifically, in the Mediterranean basin, infection cases have been reported in Greece, Turkey and Egypt, even if unconfirmed. In Italy, bacterial strains have been isolated from environmental samples of water and soils, and some infection cases in cystic fibrosis patients have been reported [60]. *Entamoeba histolytica* is instead a parasitic amoebozoan of humans and other primates, which infects principally the intestinal mucosa causing amoebiasis characterized by symptoms, such as abdominal pain, dysentery and colon inflammation. To invade the intestinal tissues and create cysts, *E. histolytica* eludes the mucin defense by secreting cysteine, a protease that digests mucus reducing its viscosity [61]. This parasite is endemic in most tropical and subtropical countries [62], being responsible for millions of infection cases every year, sometimes with fatal consequences. *E. histolytica* is mostly transmitted for ingestion of contaminated water or food, so that the high incidence of infection in tropical and subtropical areas can also been associated with poor hygienic conditions and limited medical assistance. Nonetheless, some studies reported a strong relationship between climate conditions and *E. histolytica* infections as well, even comparing different climatic regions of the same country or verifying the infection spread in different annual periods, showing a positive correlation with temperature and humidity [63]. Finally, *Pseudomonas aeruginosa* is a Gram-negative bacterium able to infect both plant and animals, including humans, and well-known for its multidrug resistance. It is considered opportunistic because the infection appears mostly in immunocompromised individuals or in subjects affected by previous pathologies, such as cystic fibrosis and others pulmonary diseases. *P. aeruginosa* typically infects the airways, urinary tract, burns, and bruises and can reach vital organs, such as lungs and kidneys with the possibility of causing serious consequences. In fact, this bacterium can degrade the respiratory mucus by the enzymatic action of mucin sulfatase and uses the mucin as sulfate source in lungs environment [64]. It is considered ubiquitous and very little is known about specific environmental factors underlying its diffusion. However, recent studies reported a strong correlation between temperature and infection frequency, with the highest number of cases in warmer regions, suggesting that risk of *P. aeruginosa* acquisition could potentially be geographically dependent [65, 66]. According to this body of evidence, adaptations against microorganisms able to elude mucosal or cellular barriers by enzymatically inactivating mucins seem to have been evolved by the ancestors of people from Southern Italy having also secondarily reduced their IgAN genetic risk.

The S_ITA cluster was also found to have adapted to substantial UV radiation thanks to optimized melanogenesis, which might have indirectly contributed to reduce predisposition to basal cell carcinoma and other types of skin cancer. Moreover, FZD/Wnt genes subjected to positive selection only in S_ITA and playing a role in melanogenesis are known to take part to the mammalian target of rapamycin (mTOR) signaling pathway as well. Overall, loci functionally related to the mTOR gene have been demonstrated to constitute a pathway highly conserved from an evolutionary viewpoint and involved in the regulation of cell proliferation, metabolic program and senescence, as well as of nutrient sensing and mitochondrial function. Such a signaling pathway was especially suggested to be able to delay age-related diseases and/or to directly influence longevity, even in the human species [67].

**Signatures of balancing selection at FoxO signaling and arachidonic acid pathways**

Genes involved in the FoxO signaling are known mainly for their association with exceptional longevity in several species, including humans and are supposed to act as sensors of stress stimuli, such as dietary restriction, absence of insulin or insulin-like growth factors [68-72]. In particular, by cross talking with the mTOR pathway, FoxO-related loci have been proved to play a crucial role in promoting cell clearance (e.g. autophagy), which represents a protective response under nutrient deprivation/starvation that is essential for organelle homeostasis [73]. Interestingly, this FoxO-mediated response is activated also against intracellular pathogens to such an extent that alterations along this signaling cascade result in increased pathogens uptake [74, 75].

Balancing selection at genes that modulate the metabolism of arachidonic acid couples with signatures of positive selection already observed at similar loci in the overall Italian population [16]. A previous study further suggested that these adaptive events might have evolved to restrict inflammation and thus confer increased resistance to pathogens, such as *Mycobacterium tuberculosis*, secondarily favoring longevity by modulating inflammatory processes and especially the metabolism of essential fatty acids [76]. In fact, the putative adaptive alleles were found to be enriched in Italian centenarians with respect to the overall population and by mitigating the inflammatory side effects of recently adopted Western diets have been proposed to represent one of the key prerequisites to develop the longevity phenotype [76].

Balancing selection was then inferred for both the Italian population clusters to have acted at additional genes belonging to the *FoxO singaling* and arachidonic acid pathways, as well as at ADCY genes that were mostly different from those exhibiting signals of positive selection in N_ITA and that participate to the *Longevity regulating* pathway as well.
